# Laminar and Dorsoventral Molecular Organization of the Medial Entorhinal Cortex Revealed by Large-scale Anatomical Analysis of Gene Expression

**DOI:** 10.1371/journal.pcbi.1004032

**Published:** 2015-01-23

**Authors:** Helen L. Ramsden, Gülşen Sürmeli, Steven G. McDonagh, Matthew F. Nolan

**Affiliations:** 1 Centre for Integrative Physiology, University of Edinburgh, Edinburgh, United Kingdom; 2 Neuroinformatics Doctoral Training Centre, School of Informatics, University of Edinburgh, Edinburgh, United Kingdom; 3 Institute of Perception, Action and Behaviour, School of Informatics, University of Edinburgh, Edinburgh, United Kingdom; 4 Centre for Brain Development and Repair, inStem, Bangalore, India; Bar Ilan University, ISRAEL

## Abstract

Neural circuits in the medial entorhinal cortex (MEC) encode an animal’s position and orientation in space. Within the MEC spatial representations, including grid and directional firing fields, have a laminar and dorsoventral organization that corresponds to a similar topography of neuronal connectivity and cellular properties. Yet, in part due to the challenges of integrating anatomical data at the resolution of cortical layers and borders, we know little about the molecular components underlying this organization. To address this we develop a new computational pipeline for high-throughput analysis and comparison of in situ hybridization (ISH) images at laminar resolution. We apply this pipeline to ISH data for over 16,000 genes in the Allen Brain Atlas and validate our analysis with RNA sequencing of MEC tissue from adult mice. We find that differential gene expression delineates the borders of the MEC with neighboring brain structures and reveals its laminar and dorsoventral organization. We propose a new molecular basis for distinguishing the deep layers of the MEC and show that their similarity to corresponding layers of neocortex is greater than that of superficial layers. Our analysis identifies ion channel-, cell adhesion- and synapse-related genes as candidates for functional differentiation of MEC layers and for encoding of spatial information at different scales along the dorsoventral axis of the MEC. We also reveal laminar organization of genes related to disease pathology and suggest that a high metabolic demand predisposes layer II to neurodegenerative pathology. In principle, our computational pipeline can be applied to high-throughput analysis of many forms of neuroanatomical data. Our results support the hypothesis that differences in gene expression contribute to functional specialization of superficial layers of the MEC and dorsoventral organization of the scale of spatial representations.

## Author Summary

Higher brain functions such as spatial cognition are carried out in specialized brain areas. Within a specialized brain area nerve cells with different functions are organized in layers and gradients. It is possible that this topographical organization reflects underlying differences in molecular organization of the brain. However, systematic comparison of the expression patterns of tens of thousands of genes at the resolution of layers and borders is challenging. Here we develop a new computational pipeline that addresses this problem. We apply this pipeline to analysis of the medial entorhinal cortex (MEC), a brain structure that is important for spatial cognition. Our analysis shows that the MEC is highly organized at a molecular level, identifies related groups of genes that might underlie functional specialization, and implicates energy-related genes in vulnerability of certain neuronal populations to neurological disorders including Alzheimer’s disease. Our computational pipeline may have general utility for high-throughput and high-resolution analysis of brain anatomy. Our results support the notion that molecular differences contribute to functional specialization of higher cognitive circuits.

## Introduction

Spatial cognition emerges from interactions between specialized neuronal populations in the hippocampal-entorhinal system [1]. The medial entorhinal cortex (MEC) is of particular importance for cognitive functions that rely on estimation of spatial position and orientation [2]. Neurons in each layer of the MEC represent distinct information, have differing connectivity, and can be distinguished by their morphological and biophysical properties [3–7]. For example, layer II has a relatively high density of neurons with grid firing fields, whereas deeper layers contain a higher proportion of neurons with firing also modulated by head direction [3, 8]. Further topographical organization is present orthogonal to cell layers along the dorsoventral axis in that the scale of spatial representations, local and long-range connectivity, synaptic integration and intrinsic electrophysiological properties all vary with dorsoventral position [9–15]. While this specialization of encoding and cellular properties is well established, the extent to which molecular specialization defines neuronal populations within the MEC or contributes to their distinct functions is not clear.

Insights into molecular substrates for topographical organization in other brain regions have been gained through large-scale analysis of differences in gene expression [16–21]. Our understanding of the architecture and functions of MEC may benefit from similar approaches. While detailed anatomical and histochemical studies have shown that certain genes, including reelin, calbindin [22] and some cadherins [23], identify cell populations associated with particular layers of the MEC, we know very little about the identity, laminar or dorsoventral organization of the vast majority of genes expressed in the MEC. This is a difficult problem to address for the MEC as its borders with adjacent structures are ambiguous, it has a dorsoventral as well as a laminar organization and its similarity to other cortical structures is unclear. As a result, key questions about its molecular organization are currently unanswered. For example, are the layers and borders of the MEC unambiguously delineated by coordinated expression patterns of multiple genes? Are genes differentially expressed along the laminar and dorsoventral axes? Do genome-wide laminar or dorsoventral differences in gene expression lead to mechanistic predictions regarding the organization of functional properties in the MEC? The MEC is ontogenetically distinct from both the 3-layered hippocampus, and from neocortex [24], with which it shares a similar laminar organization, but does this similarity to neocortex reflect a common molecular organization?

The topographical organization of the MEC extends to pathological signatures of common disorders in which it is implicated. Layer II exhibits neuronal loss [25] in patients with mild to severe Alzheimer’s disease (AD) and altered excitability in animal models [26]. Layer II is also affected in individuals with schizophrenia where there is evidence of abnormalities in cell size, organization and RNA expression [27, 28]. In contrast, epilepsy is primarily associated with loss of layer III neurons in humans [29] and in animal models [30]. Yet, the mechanisms that predispose different cell populations in the MEC to particular disorders are not known. Given the evidence of genetic associations with these diseases [31–33], it is possible that the molecular profiles of particular MEC neurons confer vulnerability. However, testing this hypothesis requires knowledge of the laminar and dorsoventral organization within the MEC of genes that are causally involved in disease.

To better understand the molecular basis for its function and pathology, we aimed to establish a genome-wide approach to define the laminar and dorsoventral organization of the MEC transcriptome. Large-scale investigations into gene expression patterns have previously used either in situ hybridization (ISH) or RNA sequencing (RNA-Seq) to identify genes with differential expression in the neocortex [16, 34], hippocampus [17] and sub-cortical structures including the striatum [20]. While transcriptomic approaches such as RNA-Seq provide a robust platform for quantification of transcripts, accurate isolation of cell populations at the resolution of cell layers is challenging [16], limiting the current applicability of this approach. In contrast, ISH is more useful for identifying patterns of gene expression because the precise location of transcripts can be examined. The Allen Brain Atlas (ABA), a high-throughput ISH database, contains brain-wide data for over 20,000 genes and has been used to identify laminar borders, and to distinguish regions and cell types in the somatosensory cortex [34], hippocampus [17], and cerebellum [35]. However, in its currently accessible form ABA data is searchable at best at a resolution of 100 µm [34] and is further limited in its utility for automated comparison of laminar gene expression because the magnitude of error in alignment accuracy of brain sections is comparable to the width of narrow individual cortical layers. Small alignment errors can therefore easily lead to incorrect assignment of genes to layers, thereby confounding systematic analysis of differences between layers.

To address these issues, we established a computational pipeline for registration and automated analysis of ISH data at a resolution of approximately 10 µm, enabling us to compare precise spatial expression patterns of over 80% of genes in the ABA dataset [34, 36]. We combined analysis of this high spatial resolution data with RNA-Seq analysis of gene expression in dorsal and ventral regions of the MEC. We demonstrate that while very few genes are uniquely expressed in the MEC, differential gene expression defines its borders with neighboring brain structures, and its laminar and dorsoventral organization. We propose a new molecular basis for distinguishing the deep layers of the MEC and provide evidence that at a molecular level deep layers of the MEC are relatively similar to those of neocortex. Superficial layers are substantially more divergent between neocortex and MEC. Analysis of genes with differential expression suggests roles in layer-specific and dorsoventral specialization of calcium-ion binding molecules, ion channels, adhesion molecules and axon guidance-related molecules. We find that differential laminar expression patterns do not extend to genes directly implicated in disease, but selective expression of related genes may provide a context that confers vulnerability to pathology in neurodegenerative diseases such as AD. Our data establish a genome-wide framework for addressing the organization of circuit computations and pathology in the MEC.

## Results

### High-resolution and high-throughput anatomical analysis of gene expression

**Figure 1:**
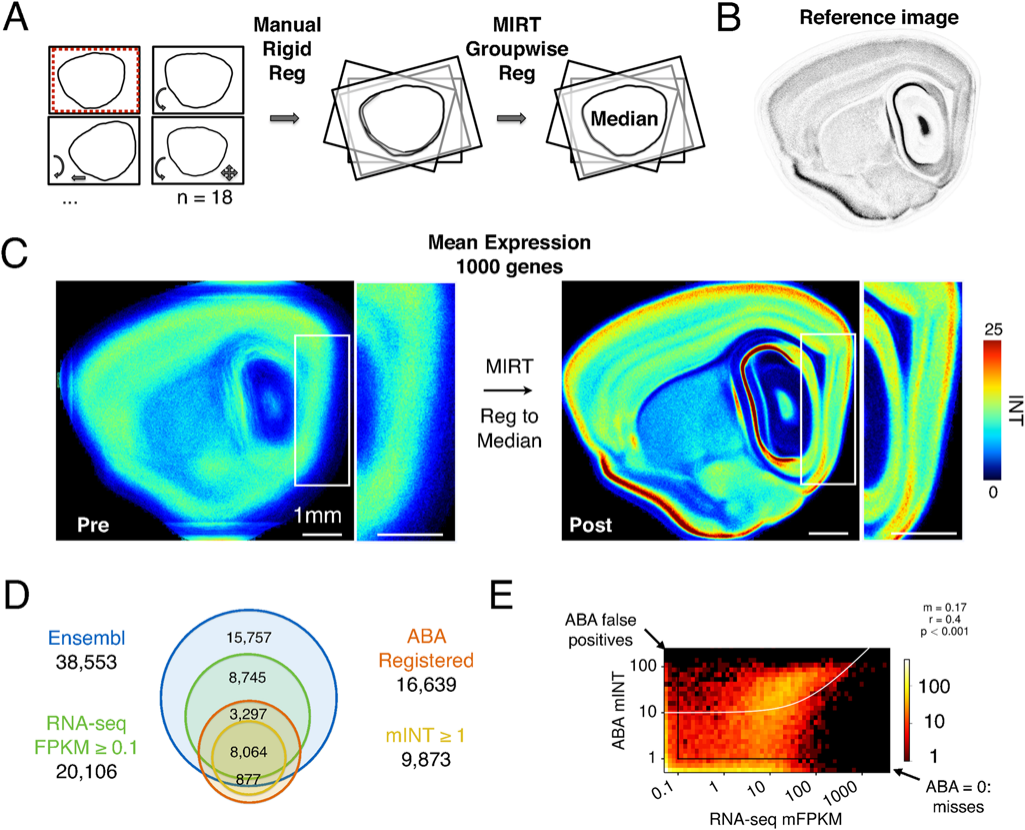
Large-scale extraction of MEC gene expression data. (A) Generation of a reference image (Im_ref_) for image warping. Images were selected (left), aligned to a template image (red dotted line) by scaling, rotation and translation (Manual Rigid Reg, centre), then registered to each other using non-linear deformation (MIRT Groupwise Reg, right). Im_ref_ was defined as the median of the resulting images (Median, right). (B) The central reference image (Im_ref_^C^, S1A Fig.). (C) Image registration reveals laminar organization of gene expression. Images show the mean pixel intensity (INT) for 1000 ABA expression images before (left) and after (right) registration of the corresponding pre-processed ISH image to Im_ref_^C^. Colors represent pixel intensity (Colormap adapted from the Matplotlib ‘jet’ colormap). White boxes outline the area corresponding to the MEC, shown at higher magnification. (D) Venn diagrams indicate the number of genes detected as expressed in the MEC using RNA-seq analysis (Ensembl v73) and/or our re-registered ABA data set. (E) 2D histogram indicating the number of genes with a particular FPKM and mINT, represented using a log scale (right). White line is the linear regression fit, which indicates that RNA-seq and ABA expression are positively correlated. Data were averaged across the dorsal and ventral region. Data with zero values are included in the first histogram bin.

To be able to systematically compare expression of genes in the adult mouse MEC at laminar resolution we extended the precision with which the localization of expressed genes in the ABA dataset can be compared. To achieve this we implemented methods to warp ISH images and their corresponding processed expression images, in which pixel intensity is used to represent the relative total transcript count [34], into a standard reference frame (see Materials and Methods, Fig. 1A-B and S1A Fig.). Our re-registered ABA data set contains at least 1 image meeting our quality criteria and that contains the MEC for 16,639 genes (81.2% of genes in the ABA sagittal dataset) (S1B Fig.). Non-linear registration provides a striking improvement in the spatial resolution at which the localization of gene expression can be compared (Fig. 1C). Prior to re-registration, exploration of the organization of gene expression is confounded by variability in the shape and size of brain sections used in different ISH experiments. For example, averaging ISH expression images for 1000 genes prior to registration results in diffuse images without any laminar organization (Fig. 1C). In contrast, following re-registration of the same images, neocortical layers are clearly distinguishable from one another (Fig. 1C). Other landmarks, for example the white matter border with the striatal region, the hippocampal pyramidal layer and layer I of the piriform cortex, also become clearly identifiable (Fig. 1C). Within the MEC, the resolution is such that multiple layers and a dorsoventral organization can be recognized. Thus, our computational pipeline for image registration enables high-resolution comparison of gene expression between layers and along the dorsoventral axes of the MEC, as well as with other brain regions.

To validate gene expression data extracted from the ABA we compared mean pixel intensity values across dorsal and ventral MEC with RNA-Seq data acquired from the same regions (see Materials and Methods, S1A Fig.). Our RNA-Seq analysis detected 20,106 of the 38,553 genes (52.7%) in the Ensembl mouse database (release 73), including 15,496 protein-coding genes. In comparison, of the 16,639 genes from the ABA dataset for which we successfully registered images, our analysis revealed that 9,873 (59.3%) are expressed in the MEC, including 8,941 genes that we could identify in the Ensembl database (Fig. 1D). Of the registered Ensembl genes, 8,064 (90.2%) were also detected by RNA-Seq, indicating a high degree of consistency between the two approaches (Fig. 1D). This is supported by a significant positive correlation between RNA-Seq transcript FPKM (fragments per kilobase of exon per million fragments mapped [37]) and mean pixel intensity of ABA images (r = 0.40, p < 2.2 × 10^-16^) (Fig. 1E). It is possible that the 877 genes that appear to be expressed in ABA data, but are not detected by RNA-Seq, are false positives, while the 3,297 genes detected using RNA-Seq that are not detectable in the ABA data, may reflect false negatives in the ISH data, for example due to errors in probe design, staining or image processing. A further 6,463 genes detected using RNA-Seq that are not in the re-registered ABA dataset (Fig. 1D) include 1,939 pseudogenes and 791 long intergenic non-coding RNAs, as well as 1,855 protein-coding genes.

Together, these data demonstrate the potential of combining advanced image processing tools for high resolution alignment and analysis of ISH data sets with RNA-Seq. RNA-Seq enables quantification of thousands of transcripts over a large dynamic range, while automated analysis of ISH data reveals gene expression at laminar resolution that can be quantified and compared within and across brain regions.

### Gene expression detected by ISH distinguishes MEC from other brain regions

Previous investigation using microarrays to compare tissue harvested from multiple brain regions has shown that gene expression in the entorhinal area is most similar to that of neocortex and hippocampus and least similar to non-telencephalic regions [38]. However, it is not clear if there are individual genes that distinguish these brain regions, whether differences show laminar or dorsoventral specificity or if this pattern applies to the MEC specifically rather than the entorhinal area as a whole.

**Figure 2:**
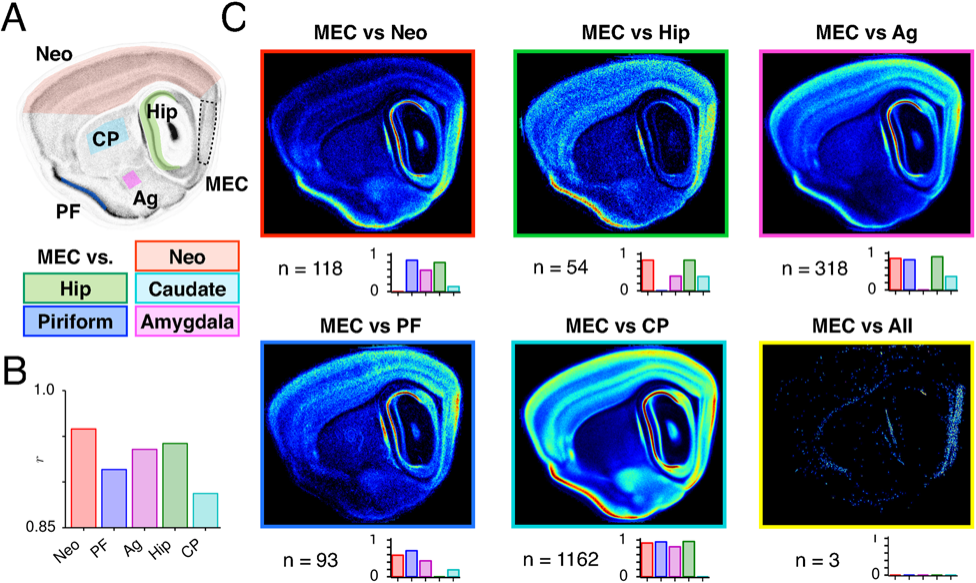
Identification of MEC-enriched genes. (A) Locations on the central reference image (Im_ref_^C^) for which mean pixel intensities (mINT) were extracted from the MEC, neocortex (Neo), hippocampus (Hip), piriform cortex (PF), caudate putamen (CP) and amygdala (Ag). (B) Bars indicate Pearson’s correlation coefficients between mINT^MEC^ and mINT^[OTHER]^ for all genes in the re-registered ABA data set. (C) Images show the average pixel intensities throughout sagittal sections corresponding to the central reference section of genes that have at least 4-fold higher mINT* in MEC than in neocortex (red frame), hippocampus (green), piriform cortex (blue), amygdala (magenta) or caudate putamen (cyan) individually. Just 3 genes are expressed at higher levels in MEC than other brain regions pooled together (yellow frame). Lower bar charts indicate the proportion of genes in each list that are expressed in each other region, given the same inclusion criteria.*Inclusion criteria: mINT^MEC^ ≥ 2 and mINT_norm_^MEC^ ≥ 0.8 (≥ 0.99 if mINT^MEC^ < 5) and mINT^OTHER^ < 5.

To first determine whether gene expression in the MEC alone shows a relationship to other brain areas similar to that established by microarray analysis, we used the re-registered ABA dataset to isolate ISH gene expression data from several brain regions (Fig. 2A). Consistent with microarray data [38], we found that gene expression in MEC correlates most strongly with neocortex (r = 0.958, p < 2.2 × 10^-16^), amygdala (r = 0.936, p < 2.2 × 10^-16^) and the hippocampal pyramidal layer (r = 0.942, p < 2.2 × 10^-16^) and more weakly with the caudate putamen (r = 0.887, p < 2.2 × 10^-16^) (Figs. 2B, S2A-B). The relatively high correlations between the MEC and the other regions suggests that differential expression of relatively few genes is likely to underlie functional differences between these areas.

To investigate whether the expression of single genes could distinguish MEC from other regions, we identified genes with at least 4-fold higher mean pixel intensity in MEC compared with each of the other regions (S2C Fig., Materials and Methods). This analysis revealed 118 genes that are expressed at higher levels in MEC than neocortex, 93 for piriform cortex and 54 for the hippocampus, compared with 318 for the amygdala and 1,162 for the caudate putamen. These numbers decrease as the threshold difference in mean intensity between MEC and the other regions is increased (S2D Fig.). Section-wide average images reveal that the expression within the MEC of these genes is often not uniform, but can be concentrated in specific layers (e.g. MEC vs neocortex) or dorsoventrally organized (MEC vs piriform cortex and amygdala) (Fig. 2C). They also reveal that genes selectively enriched in MEC compared with one region are, on average, also strongly expressed in other regions (Fig. 2C). Nevertheless, 3 genes could be identified as uniquely enriched in the MEC compared with the other 5 regions (Fig. 2C), although expression of each followed a laminar organization and did not mark the MEC as a whole. We also asked if combinations of expressed genes might better distinguish the MEC from other regions. However, we found that only in a minority of pairs of genes with converging expression in MEC (14/456) does expression fully colocalize to the same laminar and dorsoventral regions (S2E-F Fig.).

Together, these data further validate our quantification of MEC gene expression and indicate that few, if any, individual genes or pairs of genes are likely to distinguish the MEC as a whole from other brain regions. Thus, specific attributes of the MEC are unlikely to be a product of highly specific expression of a few genes. Instead, our data are consistent with combinatorial expression of larger sets of genes defining differences between cell populations in the MEC and other brain areas (c.f. [39]). Our data also highlight a limitation of regional comparison of gene expression in that genes which are co-expressed in a given brain region may not be colocalized to the same cell layer or dorsoventral area. Therefore, to better understand the laminar and dorsoventral organization of gene expression in the MEC, and the relationship between the organization of the MEC and neocortex, with which it has the most similar overall gene expression [38], we took advantage of our pipeline for large-scale comparison of gene expression to analyze expression at laminar and sub-laminar resolution.

### Differential gene expression defines MEC borders with neighboring structures

Borders of the MEC, which we consider here as the region also previously referred to as the caudal entorhinal field [4, 40, 41], have typically been defined on the basis of classical cytoarchitectonic criteria, chemoarchitecture and connectivity [11, 40, 42]. However, because these criteria don’t always converge, ambiguity exists regarding the definition and location of the borders with adjacent regions including the parasubiculum [43, 44] as well as with more ventral structures (c.f. [42, 45, 46]). We therefore sought to determine whether ISH data, and in particular our re-registered ABA sagittal data set, would enable a clearer resolution of dorsal and ventral MEC borders, which can be viewed unambiguously in the sagittal plane.

**Figure 3:**
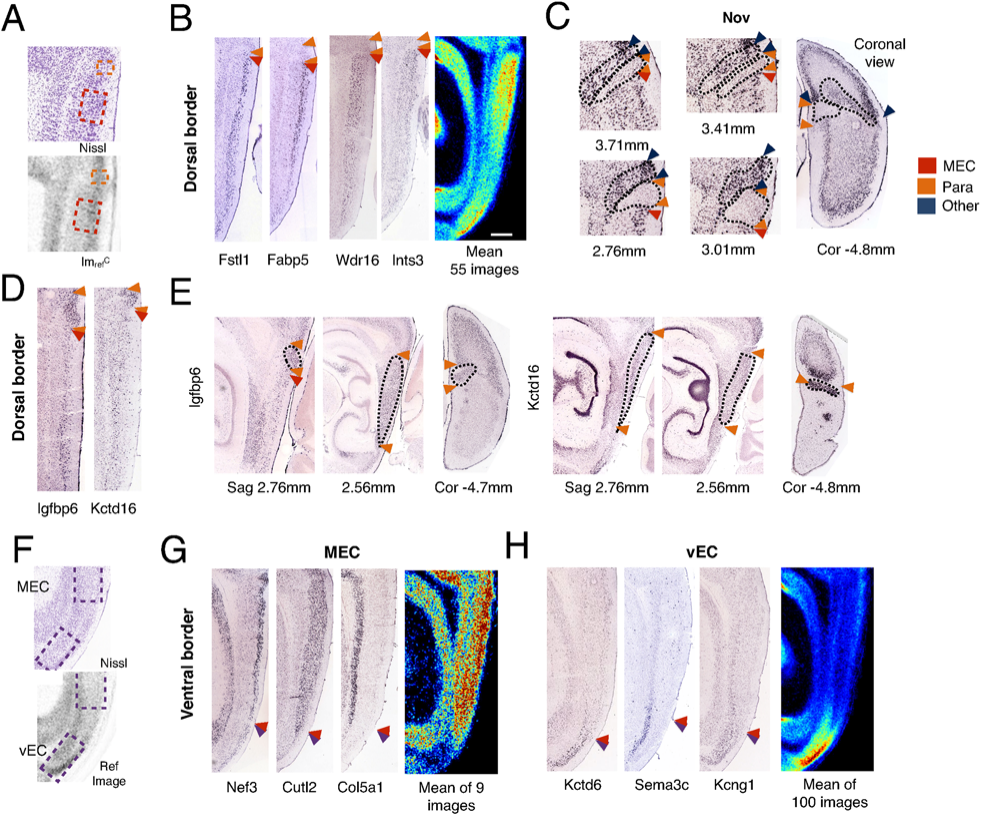
Differential gene expression defines the borders of MEC with neighboring regions. (A) Boxes indicate the dorsal (orange) and ventral (red) regions from which mean pixel intensities (mINT) were extracted for identification of genes defining the dorsal border of the MEC. (B) Example cropped ISH images downloaded from the ABA API are shown (left) adjacent to mean expression pattern for genes expressed in MEC but not the wedge-shaped region dorsal to MEC (right). Arrows indicate regional borders. (C) Raw ISH images show the dorsal border of MEC layer II marked out by the gene Nov at different medio-lateral positions (left) and in the coronal plane (right). Values in mm indicate distance from Bregma. (D) Example cropped ISH images downloaded from the ABA API are shown for genes expressed in the wedge-shaped region dorsal to MEC but not the most dorsal MEC region. (E) Raw ISH sagittal (Sag) and coronal (Cor) images from the ABA indicate the continuity of the wedge-shaped region with the more medial parasubicular region for the genes *Igfbp6* and *Kctd16*. (F) Boxes highlighting regions used to identify the ventral border of the MEC. See (A). (G-H) Example cropped ISH images downloaded from the ABA API (see Materials and Methods) are shown adjacent to images of the mean expression pattern for genes expressed in MEC but not the more ventral region (G), or in the ventral region but not MEC (H).

We focused initially on identifying genes that delineate the dorsal border of the MEC. In some atlases this region is considered as the retrosplenial or perirhinal region [47], and in others as the ectorhinal region [34, 36]. However, cytoarchitectonic, histological and electrophysiological studies in rats suggest that part of this region corresponds to the superficial layers of the parasubiculum [43, 48]. By comparing relative pixel intensity between the dorsal MEC and adjacent regions (see Materials and Methods, Fig. 3A), we identified a number of genes with expression that appears to stop at the dorsal border of the MEC (e.g. *Wdr16* and *Fabp5*) (Fig. 3B). For some of these genes, expression is only absent within a wedge-shaped region before resuming in more dorsal cortical areas (e.g. *Nov*), a pattern which is highly consistent across different medio-lateral sections (Figs. 3C, S3A-B). We therefore asked if there are genes that are expressed in the wedge-shaped region, but not the adjacent regions. We identified 9 such genes (see Materials and Methods), including *Igfbp6* and *Kctd16* (Fig. 3D). All of these genes are also expressed in the parasubicular region medial to the MEC (Fig. 3E). Of these, 7/9 also have sparse expression in superficial parts of MEC (Fig. 3D). These observations support the view that the parasubiculum extends to wrap around the dorsal border of MEC [43, 48].

At the ventral aspect of the MEC, cytoarchitectonic analysis delineates a border with the medial entorhinal field [40, 47]. We asked whether differential gene expression supports the presence of this ventral border and whether it can clarify its position. By analyzing gene expression in regions either side of the approximate location of this border (Fig. 3F), we identified genes with expression that drops off sharply (Fig. 3G, S3C-D Fig.). These genes include apparently layer-specific genes such as *Nef3*, *Cutl2* and *Col5a1* in layers II, III and V/VI respectively. We also identified genes with the converse pattern of high ventral expression and low MEC expression, including *Kctd6*, *Sema3c* and *LOC241794* (Fig. 3H). These expression patterns are consistent across different mediolateral sections (S3C-D Fig.). Thus, the ventral border of the MEC can be identified by genes with sharply increased or reduced expression.

### Differential gene expression defines MEC layers and intra-laminar organization

**Figure 4:**
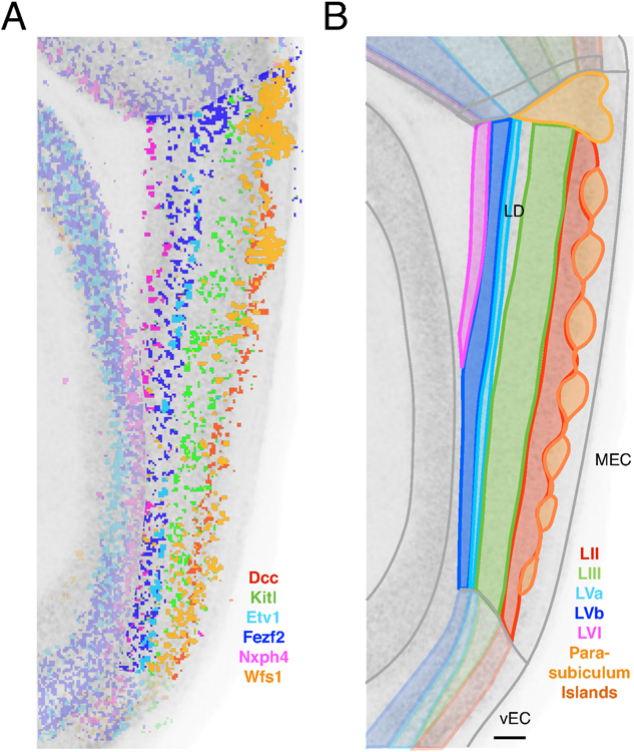
Laminar and intralaminar organization of MEC defined by differential gene expression. (A) Composite image shows high-intensity pixels for 6 exemplar genes with layer-specific expression. Pixels outside of the MEC and parasubicular region are made semitransparent. Images for the genes *Dcc* and *Wfs1* were re-registered for a second time after manual pre-processing to improve the quality of registration for this figure. (B) Schematic showing a refined genomic atlas of the layers and borders of MEC. The region between layer III and V is the lamina dissecans (LD).

Cytoarchitectonic and developmental studies indicate that the MEC is a type of periarchicortex (paleocortex), a transitional structure between 6-layered neocortex and 3-layered archicortex, with 5 cytoarchitecturally distinct cell body layers [24, 40, 45]. However, while layer II and III are easily distinguished by their cytoarchitecture and connectivity (cf. [40]), differentiation of cell populations within layers V and VI is less well established, although there is evidence that the cell bodies and dendrites of distinct cell types are differentially distributed within these layers [4, 6, 40, 49]. Layer IV corresponds to the cell free lamina dissecans [40]. To better define the laminar organization of the MEC, and to be able to compare its structure to other cortical regions, we therefore asked if differential gene expression distinguishes the superficial from deep layers or clarifies laminar borders within the deep layers (Figs. 4 and S4).

We first identified 159 genes specifically expressed in layers II, III or V/VI (see Materials and Methods and S4A-C Fig.). We refer to these genes, which show no consistent expression in other layers, as layer-specific genes (see Materials and Methods). We initially analyzed deep layers (V—VI) together because their divisions and borders are not easily distinguished by cytoarchitectonic criteria. Since genes with layer-specific expression patterns are of particular interest as neuroscience tools for isolating laminar functions, we examined their likely validity. Almost all layer-specific genes could be detected in our RNA-Seq analysis (149/159 with mean FPKM ≥ 0.1) and 62/159 had substantial levels of expression (mean FPKM ≥ 10). We also identified a further set of 622 genes, which we define as strongly differentially expressed (DE)(see Materials and Methods, S4B Fig.). These genes are expressed at higher levels in at least one layer than another, but are not necessarily exclusive to one layer. Both layer-specific and DE genes show consistent expression patterns across mediolateral sections (S6 Fig.). Only 37 of the layer-specific genes and 144 of the DE genes are amongst the 1000 most viewed genes in the ABA [50]. Thus, layers of the MEC can be distinguished by layer-specific and DE genes, many of which have received little previous attention suggesting they may represent new targets for future exploration.

Further examination of genes specific to the deep layers revealed three separate divisions of layers V and VI. First, a narrow zone at the deep border of layer IV is distinguished by expression of 5 genes, including *Etv1*, *Grp* and *Nts* (Figs. 4A, S4D). A second narrow zone of cells that is adjacent to the white matter is delineated by the expression of 8 genes, including *Jup* and *Nxph4* (Figs. 4A, S4D). Finally, the wide intervening region is distinguished by 20 genes, including *Thsd7b*, *Cobll1* and *Col5a1* (Figs. 4A, S4D). Because layer V has been suggested to have a narrow superficial and wider deep zone [40], we refer here to the two more superficial subdivisions as layer Va (narrow) and Vb (wide), and we refer to the layer bordering the white matter as layer VI [40]. This delineation of layers Va, Vb and VI is supported by patterns of expression within the deep layers for the larger set of DE genes (layer Va (n = 24), Vb (n = 55) and VI (n = 13); S4E Fig.). A further 27 DE genes are expressed in both layer Va and VI, but not Vb (S4E Fig.). Thus, patterns of gene expression enable differentiation of divisions within the deep layers.

Previous cytoarchitectonic and electrophysiological studies have indicated that within layer II a subset of cells are clustered in islands [51–54]. In mice, neurons within islands express calbindin, whereas neurons outside islands express reelin [22, 53, 54]. Cells in these islands are of particular interest as they differ from reelin-positive cells in both their electrophysiology and projection targets [22, 53, 54]. Taking all DE genes within MEC, we found 30 genes that within layer II are predominantly expressed in apparent islands (Figs. 4A, S4F). Of these, just 8 are also specific to layer II within the MEC (S4G Fig.), including the calcium-binding protein, calbindin (*Calb1*). A further 19 are strongly expressed in the MEC deep layers but not layer III. The island genes include 13 genes that are expressed in the wedge-shaped patches of presumed parasubiculum adjacent to dorsal MEC, 3 of which we identified earlier (e.g. *Mrg1*, S4F Fig.). We also identified 37 genes with the converse, ‘Inter-island’, pattern (S4F Fig.). Of these genes 23 are specific to layer II, including *Reln* (reelin) and *Il1rapl2* (S4G Fig.). A further 11 are also strongly expressed in the MEC deep layers, in particular in layer Va. The remaining layer II-specific genes do not appear to be uniquely expressed in either island or inter-island regions (S4G Fig.). Thus, differential gene expression distinguishes cell populations within layer II, shows that cells within and outside islands may be distinguished from cells in other layers by expression of common genes and provides evidence of similarities between the layer II island cells and parasubiculum.

### Deep layers of MEC and neocortex show greater molecular similarity than superficial layers

What is the relationship between laminar organization of the MEC and other regions of cerebral cortex? While MEC has greatest similarity in gene expression to neocortex and also shares a similar laminar structure, classic ontogenetic evidence indicates that the two regions are developmentally distinct [24]. However, it is unclear if these ontogenetic differences are associated with later molecular differences between specific layers of the mature cortices.

**Figure 5:**
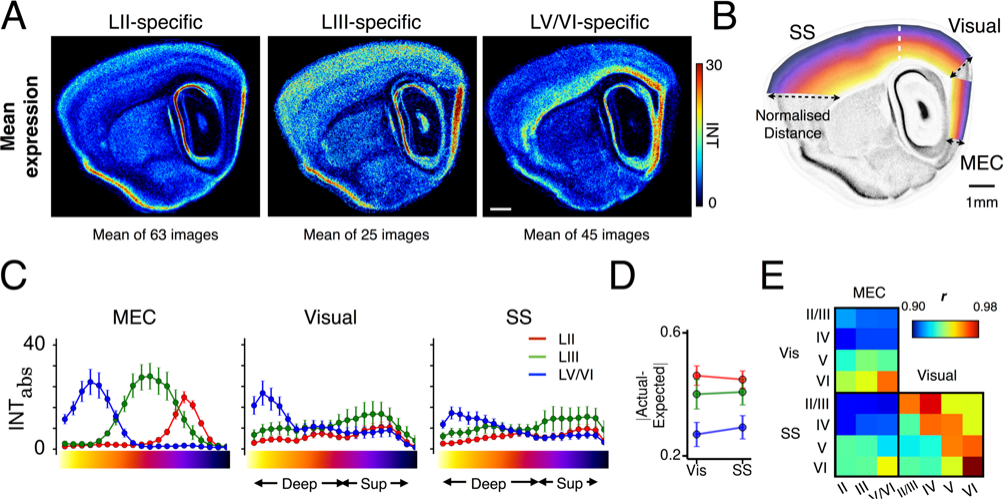
Molecular similarity between neocortex and MEC is greater for deep than superficial layers. (A) Mean expression patterns of layer-specific genes with images in the ABA re-registered data set corresponding to the central reference plane. Scale bar 1mm. (B) Schematic of the central reference image showing the MEC, visual and SS regions overlaid by a color-coded map representing the normalized distance from the inner white matter (0) to the brain surface (1). Pixel intensities were extracted from all locations and binned into 20 groups according to normalized distance. (C) Plots of the distribution of pixel intensities for each MEC layer-specific gene group (see (A)) as a function of distance from the inner white matter border. Error bars represent standard error of the mean. There is a main fixed effect of layer-specific group on neocortical expression (Mixed Model Analysis, *F* = 22, *p* < 0.001). Arrows indicate regions of deep and superficial (Sup) neocortex. Laminar boundaries were estimated using individual and mean expression profiles of MEC and SS layer-specific genes (S5 Fig.). (D) Genes with deep layer-specific expression in MEC show significantly more similar expression patterns in their equivalent neocortical layers than in superficial layers (MANOVA, Overall effect of layer-specific group: *F_(4,174)_* = 3.3, *p* = 0.012; Between-subjects effects of layer-specific group: Vis *F* = 6.7, p = 0.002; SS: *F* = 6.0, p = 0.004. Tukey’s HSD Vis: Deep < LII: *p* = 0.002; Deep vs. LIII: *p* = 0.051; SS Deep < LII: *p* = 0.04; Deep vs. LIII: *p* = 0.051). (E) Correlation matrix color represents the Pearson’s correlation coefficient (***r***) between mean pixel intensity (mINT) in particular layers of MEC, visual and SS cortices for all genes in the re-registered ABA data set.

To address this we first systematically examined overall expression in visual cortex and somatosensory (SS) cortex of genes with layer-specific expression in MEC. When we averaged expression of all genes selectively expressed in layers V/VI of MEC we found them to have a similar laminar organization in neocortex (Fig. 5A). In contrast, mean expression in neocortex of genes localized specifically to layer II or III of the MEC has a less distinct laminar organization (Fig. 5A). To quantify these differences we measured the distribution of gene expression intensity as a function of distance from the corpus callosum to the pial surface (Fig. 5B, Materials and Methods). We found that the three groups of MEC layer-specific genes have differing expression patterns in neocortical regions (Figs. 5C, S5A; Mixed Model Analysis, *F* = 12.3, *p* < 0.001). To assess the degree to which the laminar organization of each group of MEC layer-specific genes is maintained in neocortex, we first calculated the ratio of their expression in deep layers (V and VI) to superficial layers (II-IV). We then calculated the difference between these ratios and their expected values of 1 for deep genes, and zero for superficial genes. This difference was significantly smaller for deep layer-specific genes compared with superficial layer-specific MEC genes (Fig. 5D; MANOVA, p = 0.002 and p = 0.004 for effect of MEC layer-specific group in visual and SS cortex respectively). A similar relationship is apparent when we consider the expression patterns of individual genes (S5B, C, D Fig.). Around 92% of deep layer-specific MEC genes are expressed in the neocortex and 61–64% of all these genes are enriched in deep visual and SS cortex, respectively (S5C Fig.). In contrast, 77% of superficial layer genes are expressed in SS or visual cortex, but just 28–43% are enriched in superficial layers (S5C Fig.). Thus, our analysis of layer-specific genes supports the idea that deep layers of MEC have greater similarity to neocortical regions than superficial layers.

To investigate whether the relationship between layers of the MEC and neocortex extends beyond layer-specific genes, we took all genes in the re-registered ABA data set and examined the correlations in pixel intensity between layers in different cortical regions (Fig. 5E). MEC deep layers together correlate most strongly with neocortical layer VI (*r* = 0.96,0.94, *p* < 2.2 × 10^-16^), while layer II and III of MEC are more strongly correlated with neocortical layer V (*r* = 0.93–0.95, *p* < 2.2 × 10^-16^) than II or III (*r* = 0.90–0.91, *p* < 2.2 × 10^-16^) (Fig. 5E). To establish whether these correlations differ from those between neocortical regions, we investigated correlations between visual and SS cortices across all genes. We found that all corresponding layers correlated strongly (*r* > 0.96, *p* < 2.2 × 10^-16^) (Fig. 5E). Similarly, when we examined expression patterns of SS cortex layer-enriched genes [34, 36], we found a similar laminar organization of expression between SS and visual cortex (S5E Fig.). Thus, while laminar organization of gene expression is maintained between neocortical regions, gene expression within superficial layers of MEC, in particular, diverges from corresponding layers of neocortex.

Given the overall similarity between gene expression in deep layers of MEC and neocortex, we examined possible relationships between particular deep layers in each region. Of genes specifically expressed in particular deep layers we found that MEC layer VI-specific genes are almost always also expressed in layer VIb of neocortical regions (n = 7/8; Figs. 5A, S5D) (c.f. [55]). Meanwhile, layer Vb-specific genes are more commonly expressed in layer VIa of neocortex than layer V (S5D Fig., n = 15 vs 7 / 19). Moreover, MEC deep layers together correlate most strongly with neocortical layer VI (*r* = 0.96,0.94, *p* < 2.2 × 10^-16^), and more weakly with layer V (*r* = 0.94, 0.91, *p* < 2.2 × 10^-16^)(Fig. 5E). This is consistent with our observation that MEC layer Vb genes are more commonly expressed in layer VIa of neocortex than layer V, as the layer Vb region occupies the majority of the area of the MEC deep layers. Thus, at the level of gene expression MEC layers Vb and VI can be considered most closely related to neocortical layers VIa and VIb, respectively.

In summary, our analysis provides molecular evidence for an organization in which deep layers of MEC and neocortex implement similar gene expression programs, whereas superficial layers of MEC and neocortex express more diverse sets of genes.

### Excitability and communication-related genes show laminar organization

**Figure 6:**
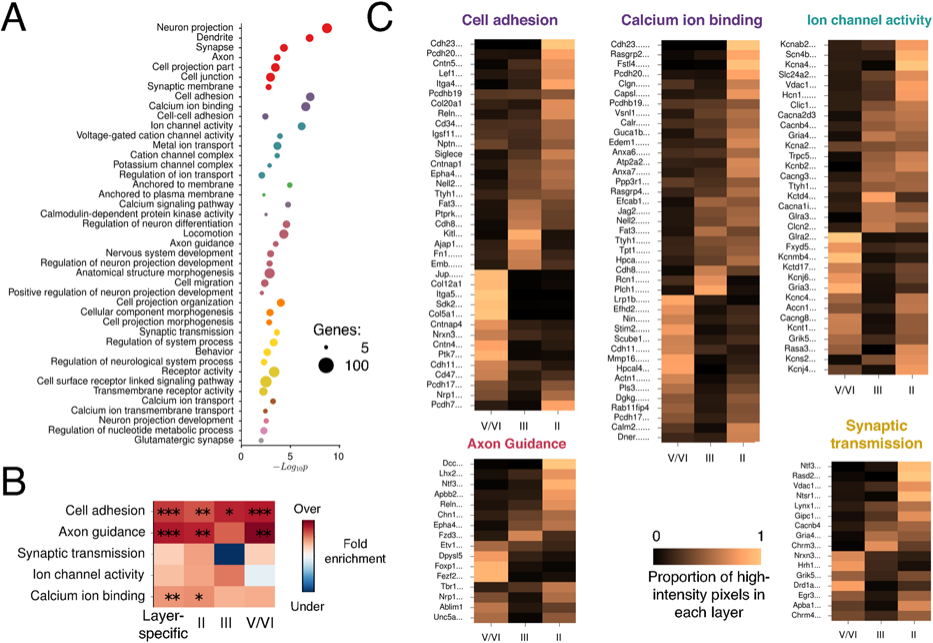
Functionally grouping of genes with laminar organization. (A) Enriched functional clusters of gene ontology (GO) and KEGG pathway terms. Overrepresented annotations were identified using an overrepresentation analysis in GOElite [102]. Colors reflect terms clustered based on kappa similarity [103]. Only the most significant annotations (FDR < 0.01) that are sufficiently different from other terms in cluster (kappa score < 0.7) are shown. (B) Colors indicate the log_2_ fold enrichment of the term in the layer-specific list, or lists for individual layers, compared with all MEC-expressed genes. Asterisks indicate significance at * α = 0.05, ** α = 0.01, *** α = 0.001. (C) Colors represent the normalized proportion of high-intensity (≥ 2 x mINT^MEC^) pixels in each layer for genes in each of the indicated overrepresented groups. Data were normalized by dividing the proportion for each layer by the sum across layers.

Our analysis suggests specialized gene expression in different layers of the MEC. If this reflects an underlying functional organization then it could be reflected in the functions associated with layer-specific or DE genes. Given known differences in electrical intrinsic properties, morphology, connectivity and organization of cells between MEC layers [4, 6, 40, 45], we hypothesized that genes involved in cell excitability and communication might be differentially expressed across layers. To test this, we focused initially on DE genes as their greater number gives more statistical power in identifying over-represented gene attributes. We identified Gene Ontology (GO) annotations and pathways that are overrepresented amongst DE genes (n = 722 Ensembl-identified genes) relative to all genes expressed in the MEC (n = 9,057 Ensembl-identified genes). To reduce redundancy and identify diverse functions of interest, we clustered enriched terms into groups. Consistent with our prediction, genes associated with neuronal projections (n = 75, *p_adj_* = 1.85 × 10^-9^), particularly synapses (n = 45, *p_adj_* = 4.57 × 10^-5^), and those involved in calcium ion binding (n = 61, *p_adj_* = 2.72 × 10^-7^), cell adhesion (n = 54, *p_adj_* = 9.48 × 10^-8^), and axon guidance (n = 23, *p_adj_* = 3.14 × 10^-4^) are overrepresented amongst DE genes (Fig. 6A). We also found strong enrichment of genes involved in ion channel activity (n = 46, *p_ad_*_j_ = 7.05 × 10^-7^) and synaptic transmission (n = 25, *p_adj_* = 2.36 × 10^-4^) (Fig. 6A). Amongst ion transport-related genes, cation channel activity (n = 34, *p_adj_* = 3.82 × 10^-5^) is particularly enriched whereas anion channel activity is not. We asked if attributes enriched among DE genes were also identifiable amongst layer-specific genes. In addition to being significantly overrepresented amongst all DE genes, cell adhesion, axon guidance and calcium ion binding-related genes were also significantly overrepresented amongst the group of layer-specific genes (Fig. 6B). Given critical roles of these genes in neuronal signaling, these data support the idea that laminar differences in gene expression within the MEC support laminar organization of computations within MEC microcircuits.

Are genes within the functional groups that are overrepresented amongst DE genes enriched in particular layers or are they distributed across layers? Comparison of expression patterns for individual genes revealed genes with enriched expression in each layer (Fig. 6C, Materials and Methods). This analysis highlights a number of genes of potential functional importance. For example, ion channel-related genes include the potassium channel subunits *Kcna4* and *Kcnmb4*, which control excitability and are enriched in layers II and Vb respectively, while axon guidance/adhesion-related genes enriched in layer II include *Lef1*, *Lhx2* and *Dcc* as well as the ephrin receptor gene *Epha4* (Fig. 6C). A possible role for the latter genes could be to control guidance of axons to newborn granule cells in the dentate gyrus [56]. Cell adhesion-related genes are also selectively expressed and significantly overrepresented in all layers and include several cadherins and protocadherins (Fig. 6B-C). These data suggest that subsets of each functional group of DE genes are expressed in each layer.

Together these data reveal candidate categories of genes that are most likely to distinguish the functions of different layers within the MEC. Our analysis also identifies molecules with highly specific laminar expression that could contribute to particular electrical and synaptic properties.

### Gene expression in MEC is systematically organized along the dorsoventral axis

Topographical organization of intrinsic features along the dorsoventral extent of the MEC has received considerable interest because the characteristics of grid cells vary systematically along this axis [9, 10, 12, 13, 57]. The extent to which gene expression parallels this organization is not currently known. We took two approaches to addressing this issue, one using our re-registered ABA dataset, with its advantage of high spatial resolution, and the other using RNA-Seq analysis, which enables quantification across a wide dynamic range and the ability to test the reproducibility of gradients. This combined approach therefore enabled us to question not only dorsoventral differences in gene expression, but also their laminar organization.

**Figure 7:**
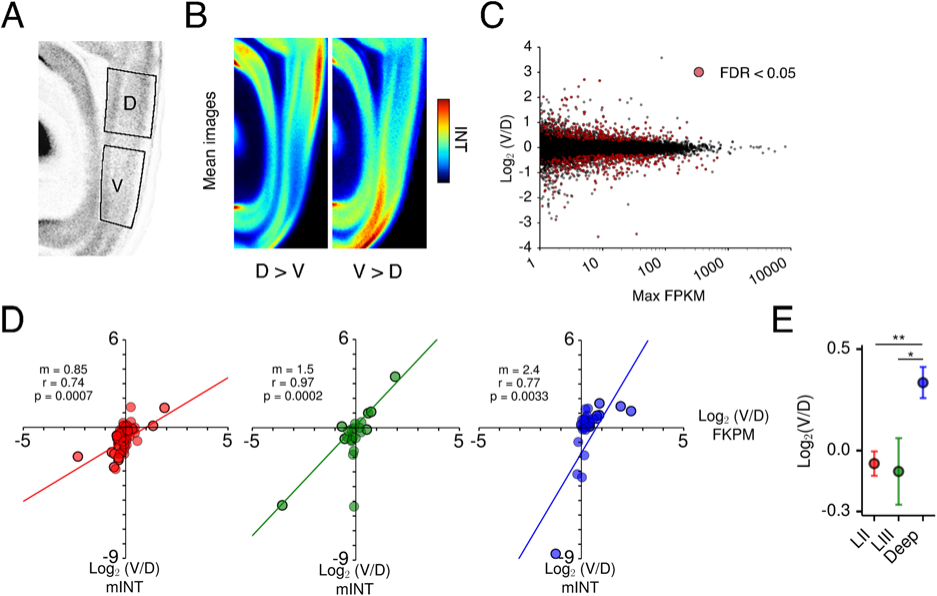
Dorsoventral organization of gene expression in MEC. (A) Boxes overlaid on MEC region of the central reference image indicate the regions from which pixel intensity was measured and a mean calculated. Pixel intensity was also measured from the adjacent lateral image if present in the re-registered ABA data set. Intensities for dorsal and ventral regions were averaged across the 2 planes to give a mean (mINT) in the dorsal and ventral regions. (B) Average images indicate mean expression patterns for genes with higher dorsal (D>V) and higher ventral (V>D) expression that have images in the central plane. Genes were classified based on the following criteria: All: mINT^MEC^ ≥ 2, D>V: log_2_(V/D) mINT ≤ -0.2630, V>D: log_2_(V/D) mINT ≥ 0.2630. (C) Identification of dorsoventrally patterned genes using Cuffdiff 2 differential expression analysis [58] of RNA-Seq data. Scatterplot shows log_2_(Ventral FPKM/Dorsal FPKM) as a function of absolute FPKM. Significant genes (FDR < 0.05) are indicated in red. Only genes with mean FPKM ≥ 1 across samples and that had a sufficient number of reads for analysis were included. (D) Scatterplots show log_2_(V/D) mINT for ABA images of genes with layer-specific expression as a function of log_2_(V/D) FPKM for corresponding RNA-Seq data points. Slopes (*m*) were obtained using a linear regression analysis. Black outlines indicate genes with FDR < 0.05 using Cuffdiff 2 analysis. (E) The ratio of ventral to dorsal expression is significantly higher for deep-layer specific genes than superficial layer specific genes (1-way ANOVA *F* = 7.47, *p* = 0.0008. Post-hoc Tukey’s HSD LII vs. Deep *p* = 0.0016, LIII vs. Deep: *p* = 0.010).

We first calculated the ratio of pixel intensity between dorsal and ventral regions in images from the re-registered ABA dataset (Fig. 7A). We defined genes with at least 20% more expression in the dorsal than ventral area as being expressed higher dorsally (D>V) and those with at least 20% more in the ventral area as being expressed higher ventrally (V>D) (see Materials and Methods, S7A Fig.). As a result, we identified 3,188 D>V genes compared with 1,352 V>D genes (Fig. 7B). We next used RNA-Seq analysis to compare gene expression from microdissected regions of dorsal and ventral MEC. This also identified genes with dorsoventral differences in their expression (Fig. 7C), of which 1,467 D>V genes and 1,198 V>D genes satisfied our criteria of 20% more expression in one of the areas than the other. Of these genes 452 and 347, respectively, had statistically significant differences in expression (Cuffdiff 2 [58]: FDR < 0.05) across 4 replicate samples (Fig. 7C).

To establish whether similar populations of dorsoventrally expressed genes are identified by RNA-Seq and in the re-registered ABA dataset, we correlated the ratio of dorsal to ventral expression determined by each method. First, to avoid confounds from genes with different expression between layers, we focused on genes expressed in only one layer. We found that measures of differential expression are strongly correlated between ABA and RNA-Seq datasets for layer II, III and V/VI (Fig. 7D, LII: slope = 0.85, *r* = 0.74, *p* = 0.0007, LIII: slope = 1.5, *r* = 0.97, *p* = 0.0002, LV/VI: slope = 2.4, *r* = 0.77, *p* = 0.0038). Second, we compared gene expression for all genes found to be significantly differentially expressed across biological replicates in RNA-Seq data. We again found a significant correlation between the datasets (S7B Fig., *r* = 0.52, *p* < 2.2 × 10^-16^).

Do dorsoventral differences in gene expression manifest differently across layers? Average images indicate that layer II has the strongest D>V pattern, while the deep layers have the strongest V>D pattern (Fig. 7B). To test this, we compared the average ratio of ventral to dorsal expression for all layer-specific genes. We found significant differences in the ventral to dorsal ratio for deep layer-specific genes compared to layer-II or III-specific genes (1-way ANOVA *F* = 7.47, *p* = 0.0008. Post-hoc Tukey’s HSD LII vs. Deep *p* = 0.0016, LIII vs Deep: *p* = 0.010), with deep layers enriched for a V>D expression pattern (Fig. 7E). Indeed, while 20.6% of layer II and 14.3% of layer III-specific genes show significant D>V expression, only 1.8% of deep layer genes do (Figs. 7D, S7C).

Together our data provide convergent evidence for systematic organization of gene expression along the dorsoventral axis of the MEC, identify dorsally and ventrally enriched sets of genes, and suggest differences in the laminar organization of dorsoventral gradients.

### Functional organization along the dorsoventral axis of the MEC

**Figure 8:**
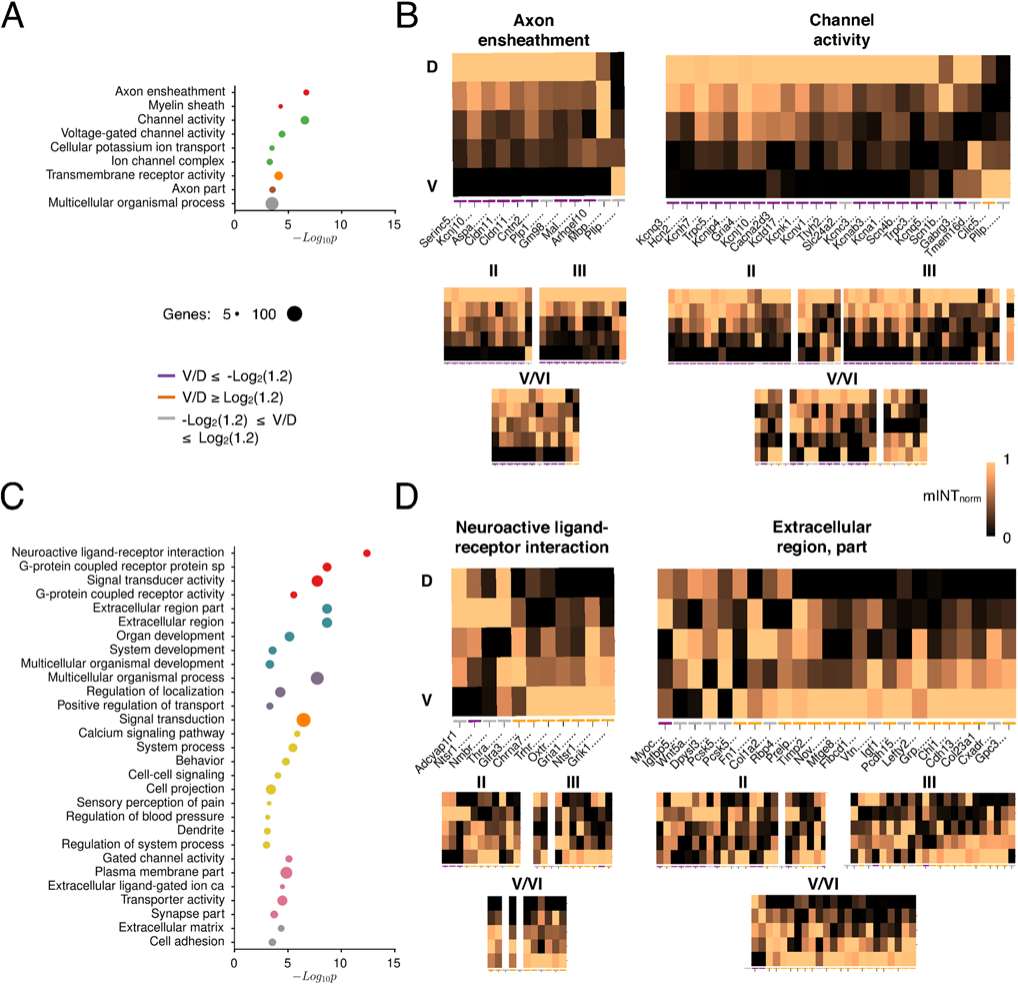
Functional grouping of genes with dorsoventral organization. (A, C) Overrepresented non-redundant GO terms for genes identified as being expressed at significantly higher levels in (A) dorsal (D>V) and (C) ventral (V>D) regions using Cuffdiff 2 analysis of RNA-Seq data [58]. Terms were clustered based on kappa similarity [103]. Only the most significant terms p < 0.001 from each cluster that are sufficiently different from each other (kappa score < 0.7) are shown. (B, D) Genes of functional interest show corresponding patterns of (B) D>V and (D) V>D expression in ABA data. Heat plots indicate the normalized mean pixel intensity (mINT_norm_) for a gene across all MEC layers as a function of dorsoventral position. Normalization was performed by setting all values to the range [0, 1]. All RNA-Seq defined D>V and V>D genes with mINT^MEC^ ≥ 2 in the re-registered data set are included. Colored bars beneath heat plots indicate whether the difference between dorsal and ventral regions was at least 20% (see legend). Plots also shown for individual layers (see S4 Fig.); data not shown for those with mINT^[layer]^ < 2. Where ISH was performed with multiple probes for one gene, data from both are shown. SP: signaling pathway.

Are the roles of genes with differential dorsoventral expression related to the cellular and system-level organization of function in the MEC? Taking all significant D>V and V>D genes identified by RNA-Seq, we investigated their possible functions using a GO and pathway analysis. By using clustering to distinguish enriched terms into key groups of interest (see Materials and Methods), we found that D>V genes are enriched for a number of attributes, particularly axon ensheathment (n = 15, *p_adj_* = 2.08 × 10^-7^) and channel activity (*n* = 32, *p_adj_* = 2.81 × 10^-7^) (Fig. 8A). We next used the re-registered ABA data set to examine the expression patterns of the identified gene groups. The D>V pattern found using RNA-Seq is replicated for the majority of axon ensheathment- (n = 9/12) and channel activity-related genes (n = 19/24) that show expression in the re-registered ABA dataset (Fig. 8B). We then investigated the layers in which gradients are strongest. Axon ensheathment genes show consistent D>V gradients in the superficial (n = 11/12) and deep (n = 9/12) layers (Fig. 8B). In contrast, genes involved in channel activity are more likely to show D>V gradients in the superficial (n = 22 / 24) than in the deep (9/24) layers (Fig. 8B).

In contrast to D>V genes, genes with V>D expression in the RNA-Seq data set are most strongly enriched for the neuroactive ligand-receptor pathway (n = 24, *p_adj_* = 4.04 × 10^-13^), which is related to G-protein coupled receptor activity (n = 35, *p_adj_* = 2.2 × 10^-9^), and for the extracellular region (n = 44, p_adj_ = 2.16 × 10^-9^) (Fig. 8C). Of the 11 identified neuroactive ligand-receptor pathway genes that are expressed in our re-registered ABA data set, 7 have consistent overall V>D patterns in the ABA (Fig. 8D) while 3 show no detectable expression. A total of 15/24 extracellular region-related genes are consistent with ABA data (Fig. 8D), with a further 12 showing no detectable expression. A possible reason for the discrepancies between ABA and RNA-Seq measures is that in the ABA analysis of the overall V>D gradient, V>D gradients that are only present in the deep layers may go undetected. This is because in some ABA images, ventral deep layers become narrower towards the medial border of the MEC and therefore ventral gene expression may be overshadowed by expression in the superficial layers or may not be present in the image. In support of this, we found that most V>D gradients found using RNA-Seq could be detected in ABA data when gene expression was specifically measured in the deep layers (Neuroactive: 8/11, Extracellular: 20/24, Fig. 8D).

### MEC vulnerability to disease

Pathological changes in the MEC have been observed in a number of neuro-developmental and neurodegenerative disorders. Whereas layer III appears to be most consistently affected in epilepsy patients and in animals models of epilepsy [29, 30], cell number and disrupted organization within layer II are consistently reported in Alzheimer’s disease (AD) [51, 59], as well as in Huntington’s (HD) and Parkinson’s disease (PD) [60], schizophrenia [61] and autism [62]. This laminar specificity suggests that particular features of these layers, whether genetic or network-based, hard-wired or experience-driven, confer vulnerability. One possibility is that genes with mutations causally linked to particular disorders have layer-enriched expression. Alternatively, broadly expressed causal genes might cause specific pathology in layers with enriched expression of genes that confer vulnerability.

**Figure 9:**
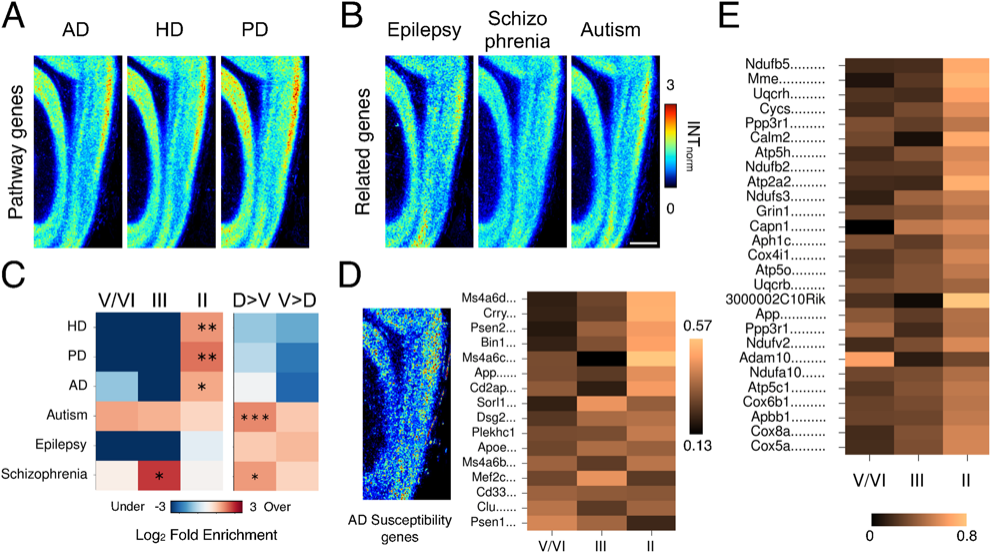
Laminar organization of disease-related molecular pathways. (A-B) Expression patterns of disease-related genes. Images show mean normalized pixel intensity (INT_norm_) across all genes that are members of KEGG pathways associated with the indicated disorder (A) and genes linked to the indicated diseases (B). Pixel intensity values for each image were normalized to the mean pixel intensity of the MEC region prior to averaging. Scale bar 500µm. (C) Colors indicate log_2_ fold enrichment of disease associations amongst layer-enriched genes (Number of enriched genes: LII:288, LIII:63, LV/VI:152) (left) and amongst RNA-seq Cuffdiff 2-measured [58] significant (FDR < 0.05) D>V and V>D genes (right). Asterisks indicate FDR < 0.05 after a two-way Exact Fisher’s test followed by Benjamini-Hochberg correction for multiple testing. Schizophrenia-related genes are overrepresented amongst layer III-enriched genes (Log_2_ Fold Enrichment = 2.22, *p_adj_* = 0.025) and both schizophrenia and autism-related genes are overrepresented amongst D>V genes (Schiz: 1.34, *p_adj_* = 0.013, Aut: 1.56, *p_adj_* = 6.1 × 10^-4^). (D) AD susceptibility genes do not show laminar organization. Averaged image for AD susceptibility genes (left) [68–71] and heat maps representing the distribution of laminar expression (centre). Colors indicate the relative proportion of high-intensity (≥ 2 x mINT^MEC^) pixels in each layer. (E) AD pathway genes show dominant layer II expression. Heat map colors indicate the relative proportion of high-intensity (≥ 2 x mINT^MEC^) pixels in each layer for genes with at least moderate laminar enrichment.

To address whether normal adult gene expression in the mouse provides insight into vulnerability, we first explored the laminar expression patterns of genes involved in signaling pathways that are disrupted in disease. Images showing the average expression pattern of genes involved in KEGG neurodegenerative disease pathways (AD, HD and PD) [63] indicate high expression in layer II, particularly in dorsal regions (Fig. 9A). To test whether this reflects significant enrichment of neurodegenerative disease pathway genes in layer II, we took all DE genes that exhibit high expression in layer II (see Materials and Methods) and compared representation of disease-related genes to their representation in the MEC as a whole. We found that AD, PD, and HD pathway genes are all overrepresented amongst layer II-enriched genes (Fig. 9C, Exact Fisher Test with Benjamini-Hochberg correction: Log_2_ Fold Enrichment > 1.19, p_adj_ < 0.024). Thus, basal gene expression may confer vulnerability of layer II in neurodegenerative diseases.

In the absence of KEGG pathway information, we used several database resources to identify genes related to schizophrenia [64, 65], autism [66] and epilepsy [67] (see Materials and Methods). Average images reveal weak, if any laminar organization for schizophrenia and epilepsy-related genes, with some evidence of layer II enrichment for autism-related genes. However, an enrichment analysis shows that schizophrenia-related genes are overrepresented amongst layer III-enriched genes (Fig. 9C), while both autism- and schizophrenia-related genes are enriched amongst RNA-Seq defined D>V genes (Fig. 9C), suggesting that pathology related to these diseases could show dorsoventral differences.

Since layer II enrichment of AD pathway genes corresponds with layer II vulnerability to AD, we explored whether genes with variants that have been established to confer increased risk of AD show layer-specific expression. AD possesses several key genetic risk factors, namely *APP*, *PSEN1*, and *PSEN2* [68], but meta-analyses of genome-wide association studies have also shown that *ApoE*, *ABCA7*, *Clu*, *Bin1*, *Cd33*, *Cd2ap*, *Epha1*, *Ms4a6A-E*, *Picalm*, *Sorl1*, *Ptk2b*, *NME8*, *FERMT2*, *CASS4*, *Inpp5d*, *Dsg2*, *Mef2c* and *Cr1* are strongly associated with late-onset AD [69–71]. We found that 16 out of the 20 of these genes that are in our re-registered ABA data set are expressed in the MEC. However, none are specifically expressed in layer II and only a minority show strong differential expression across layers (Fig. 9D). Pathology in layer II is therefore unlikely to be the result of layer-specific expression of AD risk genes. It could instead reflect enriched expression of signaling pathways linked to neurodegeneration in AD (Fig. 9A). Indeed, further analysis of the laminar expression patterns of AD pathway genes (Fig. 9E, Materials and Methods) reveals that almost all those with moderate laminar enrichment show highest expression in layer II, and that many (n = 16 / 26) are mitochondrion-associated genes, suggesting that cells in layer II may have higher energy demands than cells in other layers. This is consistent with the strong cytochrome oxidase staining observed in layer II [48]. Given that mitochondrial dysfunction is a feature of neurodegenerative disease [72] and that genes related to metabolism are altered in the MEC of patients with mild cognitive impairment [73] and AD [74], layer II vulnerability could be due to or compounded by enriched expression in layer II of signaling pathways that confer vulnerability to AD pathology.

## Discussion

To investigate the molecular organization of the MEC we combined a new pipeline for large-scale comparison of gene expression at high spatial resolution with RNA-Seq analysis. We show that differences in gene expression define the dorsal and ventral borders of MEC, its layers and its dorsoventral organization. We find that the MEC is closely related to neocortex through gene expression. This similarity is strongest for the deep layers, whereas superficial layers appear more specialized. Enriched topographical organization of genes related to synaptic communication and excitability indicates that laminar and dorsoventral organization of spatial coding within the MEC may have specific molecular substrates. Identification of laminar organization of AD-related pathways, but not risk genes, suggests that specific layers of the MEC may be particularly vulnerable to triggers of pathology in AD and other neurodegenerative diseases. The data sets generated by our study are a new resource for investigating molecular substrates for spatial coding and computation by the MEC and the structures with which it interacts, while the computational pipeline we have developed may have general applications for neuroanatomical investigation requiring comparison of many probes at high spatial resolution.

### Gene expression defines laminar organization of function and pathology within the MEC

By developing a pipeline for automated comparison of brain sections at 10 µm resolution we were able to identify genes whose expression pattern delineates the borders and layers of the MEC (Fig. 3 and Fig. 4). Validation of this pipeline against RNA-Seq data indicates that relative expression levels estimated with the two approaches are consistent (Fig. 1 and Fig. 7). Recent work using double ISH labeling validates the layer-specific expression patterns we find for cadherins in the MEC [23], while other well-characterized genes such as reelin and calbindin [22, 75] also have expected expression patterns. Our analysis identifies a further 767 genes with layer-specific or enriched expression and 799 genes with dorsoventral expression. Nevertheless, our current analysis is limited by the availability of genes in the ABA data set (20,495 / 38,553 Ensembl genes, most of which are protein-coding), by the likelihood of false negative data in the ABA ISH data where true gene expression has been missed (estimated 3,297 / 14,054 by comparison with RNA-Seq data) and by limitations in image processing and registration accuracy that prevent us making use of the entire ABA data set. While our analysis is restricted to sections in parasagittal planes containing the MEC, it could be extended to include other brain regions through additional planes and to other species including humans [76]. In principle our approach could also be extended to analysis of images from three-dimensional datasets obtained using different methods [77, 78].

Our results resolve dorsal and ventral borders of the MEC, provide molecular evidence for laminar divisions of its deep layers, and identify numerous new molecular markers for the well-established separation of the superficial layers. While dorsal and ventral borders can be distinguished unambiguously in sagittal images, medial and lateral borders are better resolved in horizontal sections, so demarcation of these borders may require use of additional horizontal data sets. Delineation of deep layers is of particular interest as they are believed to relay hippocampal output to neocortex (c.f. [4]), but their organization and functional properties have received relatively little attention. We distinguish a narrow region deep to the laminar dissecans as layer Va, consistent with that described by [45]. We also identify a distinct division of the deeper layers into Vb and VI, a narrow region of cells that appears continuous with neocortical layer VIb (Fig. 4). We suggest that the divisions previously reported within layer V [6, 40, 79] correspond to the superficial layer Va and a deeper layer Vb that we identify here. Definitive laminar delineations within the deep layers will require analysis of shared gene expression, dendritic morphology and axonal connectivity. Our results also identify new markers for island cells and, to our surprise, suggest their similarity to neurons in the parasubiculum. It will be interesting to establish whether this similarity extends to functional properties [43].

Our analysis and data sets provide a resource for future functional investigation of laminar organization of functions in the MEC. This includes identification of markers for distinguishing cell populations (Fig. 4), particularly for layer III and the deep layers, for which there are currently few specific markers. Our delineation of layers Va, Vb and VI identifies several genes in each layer whose promoters may be usable for generation of driver lines to target that cell population. We also identify common expression patterns between MEC and neocortex that may underlie shared functional roles (Fig. 5). For example, the cortical layer VI-specific immunoglobulin heavy chain gene, TIGR accession TC146068 [34], also shows expression in MEC layer Vb. It is unlikely that this similarity in expression between deep layers of MEC and neocortex reflects biased selection of exemplar genes [80] as it is present when considering all expressed genes as well as those with laminar selectivity (Fig. 5). Instead, our analysis demonstrates that deep layers of MEC show greater similarity to corresponding cortical layers than do more superficial layers. Because our analysis includes the majority of protein-coding genes (Fig. 1), it also leads to novel predictions about gene expression underlying specialized function. As well as identifying candidates for electrophysiological differences between neurons from different layers [6], many cell adhesion and axon guidance molecules are enriched amongst patterned genes (Fig. 6). Of particular interest are cell adhesion-related genes such as *Cdh13*, *Lef-1* and *Dcc* that show a similar expression pattern to *Reln*, which marks the subset of excitatory layer II cells that project to the dentate gyrus [22]. One possibility is that these genes play roles in forming connections with new born granule cells.

Genome-wide views of cortical organization can inform investigation of disease mechanisms by identifying convergent expression of molecular components of disease pathways [34, 81]. We found no evidence of layer-specific expression of genes causally implicated in disease pathology (Fig. 9). Instead, our analysis suggests that differential gene expression may underlie layer-specific pathology by predisposing specific cell populations to disease-causing mechanisms. For example, enriched expression of energy-related genes may reflect susceptibility of this layer to degeneration in AD. These functional and pathological predictions should be testable in future experimental studies.

### Dorsoventral organization of gene expression identifies molecular candidates for differences in grid scale

The dorsoventral organization of the resolution of grid firing fields and the corresponding organization of excitable and synaptic properties of layer II stellate cells [9, 10, 12, 13] suggests that cellular mechanisms for grid firing may be identifiable by comparison of key features of dorsal and ventral MEC circuits. However, until now there has been little evidence for molecular differences that could underlie this organization (cf. [12]). We provide converging evidence from re-registered ABA data and from RNA-Seq data for systematic coordination of gene expression along the dorsoventral axis of the MEC. Consistent with key roles of superficial layers in the generation of grid fields, D>V gradients were most often found in layer II and III (Fig. 7). We also found evidence for genes with the opposite V>D pattern of expression, but these were most prominent in deeper layers, suggesting that control of dorsoventral differences by molecular pathways differs across layers. A potential caveat of our analysis is that dorsoventral differences in gene expression could reflect differences in the proportions of certain cell types. While approaches such as transcriptomic analysis of isolated cells will be required to resolve this, our finding that many layer-specific genes are not significantly differentially expressed along the dorsoventral axis, while dorsoventral genes have continuous rather than all or nothing changes in intensity (Fig. 8), argues for gradients reflecting coordination of gene expression levels within populations of a single neuron type.

By taking a genome-wide approach to differences in gene expression we obtained unbiased estimates of gene functions that are enriched among dorsoventral genes. Strikingly, we found enrichment among genes with higher dorsal expression of axon ensheathment and ion channel activity (Fig. 8). This is in accordance with previous evidence for dorsoventral differences in synaptic transmission and ionic conductances [12, 13, 15], and in immunolabelling for myelin [48]. Enrichment of 10–20 genes associated with each function indicates that the corresponding cellular differences may involve coordinated control of gene expression modules. For example, our analysis extends candidates for dorsoventral differences in excitability from HCN and leak K^+^ channels [12], to include non-selective cation channels such as *Trpc5* [82] and voltage-dependent potassium channels such as *Kcnq3* [83] and *Kcnk1* (Twik1) [84]. Similarly, we identify myelin-related genes such as *Mbp* and *Plp1*, as well as related adhesion molecules such as *Cntn2 (Tag-2)*, as candidates for dorsoventral differences in coordination of axon ensheathment [85]. Future gene manipulation studies will be required to establish causal roles of these genes in dorsoventral tuning of cell properties and of spatial firing. They may also provide insight into the role of topographic gene expression in the development and maintenance of topographical connectivity between the MEC and hippocampus. Additional investigation will also be required to establish whether dorsoventral coordination of transcription is complemented by similar coordination of translational and post-translational mechanisms.

### Specialization of cortical circuits for spatial computation

Neurons in the MEC encode representations of space [9] that are critical for spatial learning and memory [86]. An unresolved question is whether this computation requires a specialized cortical circuit, or whether it is an example of a generic computation to which canonical cortical circuits can easily be adapted. Evidence for the former comes from findings that in layer II, which contains the highest density of cells with grid firing fields, excitatory stellate cells are only able to communicate indirectly via inhibitory interneurons [87–89], whereas in other cortical regions excitatory layer II principal neurons synapse with one another [90]. Consistent with this view our molecular analysis suggests considerable divergence between superficial layers of MEC and neocortex. In contrast, deeper layers of MEC appear much more similar to neocortex. Together with the dorsoventral organization of ion channel and axon ensheathment genes, our findings suggest that specialization important for spatial circuits is particularly striking within the superficial layers of the MEC. The functions within the MEC of the individual genes and functional gene groups that we identify as having laminar and dorsoventral organization have for the most part not been investigated and likely will be important targets for future exploration.

## Materials and Methods

### Ethics statement

All animal experiments were carried out according to guidelines laid down by the University of Edinburgh’s Animal Welfare Committee and in accordance with the UK Animals (Scientific Procedures) Act 1986.

### RNA-Seq data collection and analysis

Brains were rapidly extracted from 13 male 8-week-old C57Bl/6J_olaHsd_ mice and maintained in modified oxygenated artificial cerebrospinal fluid (ACSF) of the following composition (mM): NaCl 86, NaH_2_PO_4_ 1.2, KCl 2.5, NaHCO_3_ 25, CaCl_2_ 0.5, MgCl_2_ 7, glucose 25, sucrose 75), at approximately 4ºC. One 400 µm thick sagittal slice containing the right MEC was cut from each brain using a Leica Vibratome VT1200 system [91]. Dorsal and ventral regions were microdissected under a dissection microscope (S1A Fig.), with care taken to avoid inclusion of ventral entorhinal cortical regions, parasubicular or postrhinal regions and subicular regions. Tissue sections were collected into separate RNase-free eppendorf tubes before being quickly frozen on dry ice. Frozen tissue was stored at -80ºC for several weeks before RNA extraction.

We compared RNA from dorsal and ventral MEC of 4 groups of mice. To minimize the effects of inter-animal variability and variability in the dissection, whilst maintaining sufficient power to detect dorsoventral differences, samples were pooled with 3 or 4 mice in each group. RNA was extracted using RNeasy Lipid Tissue Mini Kit (Qiagen Cat:74804). RNA integrity was assessed using a Agilent 2100 Bioanalyzer. All sample RINs were between 7.1 and 8.5. cDNA was synthesized and amplified using the Ovation RNA-Seq System V2 (NuGEN Cat:7102) using 120 ng of starting material for each sample. The samples were fragmented and sequenced by the Ark-Genomics facility using Illumina HiSeq with multiplexed paired-end analysis on two lanes. Raw data were processed using Casava 1.8. Sequenced fragments were aligned using TopHat v2.0.8. After sequencing and alignment, absolute RNA expression and differential expression were computed using Cuffdiff 2 software on the output BAM files [58]. We chose Cuffdiff 2 to ensure accurate counting of transcripts in the presence of alternatively splicing. Reported gene expression therefore reflects the summed expression of all transcripts/isoforms of a gene. Cuffdiff 2 was run on the Edinburgh Compute and Data Facility (ECDF)[92] cluster on 4 cores each with 2GB of RAM. The reference genome used was Ensembl 73, downloaded 12th Nov 2013.

Transcripts were classified as expressed if their mean fragments per kilobase of exon per million fragments mapped (FPKM) [37] across samples ≥ 0.1 (c.f. [16]) in at least one of the dorsal or ventral regions (Fig. 1D). We also only considered transcripts for inclusion if Cuffdiff 2 analysis revealed them to have a minimum number of 10 alignments in a locus (default value). Transcripts were only tested for differential expression if mean FPKM across samples ≥ 1.

### ABA image processing and data extraction

The steps for processing and extraction of data from ABA images are summarized in S1 Fig. and described in detail below. Code used in this section is available at https://github.com/MattNolanLab/Ramsden_MEC.

**Image download from ABA.** Images were downloaded from the ABA database using the application programming interface (API: http://www.brain-map.org/api/index.html). Since the ABA sagittal reference atlas begins at 3.925mm laterally, and as the MEC is located between approximately 3.125 and 3.5 mm laterally, images between 0 and 1400 μm (refers to distance from most lateral point) were selected for download for each image series. Two files were downloaded for each image: an ISH image file and a corresponding expression image file. Images were downloaded using the API files: http://www.brain-map.org/aba/api/imageseries/[enterimageseries].xml and http://www.brain-map.org/aba/api/image?zoom=3& top=0& left=0& width=6000& height=5000&mime=2&path=[path specified in xml file]. Images were approximately 500KB each. Approximately 120,000 images were downloaded in total and they were stored on a cluster provided by ECDF [92].

**Preprocessing and cerebellar segmentation.** ABA images, of variable dimensions, were first downsized by a factor of 1.25 and pasted onto the center of a new image of 1200 (width) x 900 pixels (height) using the Python Image Library. ISH images were then processed to improve image segmentation and registration (S1A Fig.: steps 2–5). No further changes were made to the expression image files until application of a segmentation mask (step 6).

Image preprocessing proceeded as follows. (a) Background subtraction was carried out on the ISH images using ImageJ [93](S1A Fig.), with radius set to 1 pixel as this is approximately the size of a cell at the chosen resolution. (b) Images were thresholded using the ImageJ ‘Min_error’ automatic thresholding method such that all visible objects in the image, including anatomical features and cells with very low staining, were retained. The aim of this step was to minimize gene expression-specific information in the images whilst retaining anatomical detail to facilitate image registration based on landmark features. (c) To aid feature extraction, a smoothing filter (ImageJ) was applied to the images to smooth them prior to processing.

Because the cerebellum could impair performance of the registration algorithm we developed an automated segmentation workflow to remove the cerebellar region from images prior to registration (S1A Fig.). (d) An edge detection algorithm was applied to background-subtracted images (FeatureJ Edge detection [94]). This image was thresholded to provide two outlined regions: the forebrain and cerebellum. These regions occasionally featured internal gaps caused by very low pixel intensity brain regions. We used an ImageJ algorithm to identify the two regions as objects (defined by the complete perimeter) and to fill in any such gaps (ImageJ/Process/Binary/Fill Holes). These regions could then be detected as separate objects, using the ImageJ particle analysis tool (ImageJ/Analyze/AnalyzeParticles), and only the largest object, corresponding to the forebrain, was subsequently included in the segmentation mask. This mask could then be applied to the ISH and corresponding expression images.

Segmentation failed for images with low ISH labeling (because of edge detection failures), where the cerebellum and forebrain overlapped (due to mounting errors), and where erroneous staining prevented typical boundary detection. It was not feasible to examine all images and check those in which segmentation had failed, so we developed a method for automatically detecting successful segmentation. For each image within an image series, we used a binary support vector machine (SVM) classifier with a linear kernel to classify image masks based on success. To classify images, it is first necessary to extract features of the image that represent the patterns found in them. We used the VLFeat toolbox in Matlab [95] and custom-written code [96] to extract scale invariant feature transform (SIFT) features [97] from the images. The toolbox extracts SIFT features at 4 different scales to provide a spatial histogram that contains information about the positioning of features in space (PHOW features). For each image a feature histogram containing 4000 values was generated for input to the classifier. The SIFT feature library was provided by [96]. We trained the SVM classifier on PHOW feature vectors from 800 correctly segmented images and 250 poorly segmented images and used a further 800 positive and 250 negative images for validation and tuning of the regularization parameter. The classifier was able to separate positive and negative validation images with over 98% accuracy with a tuned regularization parameter. We therefore used the SVM model with the same parameters to obtain a score for all remaining (~120,000) images that estimated their chance of success. The majority of images were assigned positive scores but we flagged any image with a score below 1 (11% of images) as being potentially erroneous.

**Generation of reference images.** To enable the extraction of information from 2D ISH images with precision, we generated reference images for five planes covering the medio-lateral extent of the MEC and its borders (S1A Fig.). The central image (C) was our primary data extraction image, images in the adjacent lateral plane (L1) supplemented this information, while images in more lateral (L2) and in medial (M1 and M2) planes were used for reference but not for data extraction.

Reference images were generated using hand-selected ISH images that were chosen based on (1) relatively uniform expression in the MEC, (2) good tissue quality, and (3) medium ISH staining intensity. Approximately 15–20 images were chosen for each of the 5 reference images (See Figs. 1A, S1A). Pre-processed images were rigidly aligned using an ImageJ plugin “Align Image by Line ROI” (http://fiji.sc/Align_Image_by_line_ROI) [93]. Given images in which the user has marked 2 corresponding points on each image, this plugin finds an optimal transformation (translation, rotation, scale) in closed form that aligns the images into the same location.

Images then underwent group registration using a Matlab library, the Medical Image Registration Toolbox [98] (Fig. 1A). Images were registered by two-dimensional non-linear deformation to one another, with the aim of finding the group match with the greatest similarity. We chose to group register 15–20 images to capture a sufficient degree of variance without requiring excessive memory (∼ 5GB RAM) or time (∼ 20 hours). A Gaussian filter with a window size of 13 and a standard deviation of 3 was applied iteratively three times to each, followed by contrast enhancement, to enhance image structures at the relevant spatial scale. We chose to use cubic β-splines to represent the possible class of transforms and mutual information as the similarity measure, as it is relatively resistant to differences in contrast. The output of group registration is a series of transforms that correspond to each image. We generated reference images by applying these transforms and then calculating the median of the transformed images (S1A Fig.).

**Classifying images based on their medio-lateral location.** The images downloaded from the Allen Brain Atlas could be assigned either to one of the five reference image groups or to a sixth group for images not containing MEC. To ensure the ~120,000 images were appropriately classified based on medio-lateral extent, we used classification to identify, for each image series, the image most similar to our central reference image, Im_ref_^C^. We used a Support Vector Machine (SVM) library for Matlab [95] with a linear kernel and binary classification. The SVM provided a score for each image across all image series reflecting the chance that the image was approximately in the same medio-lateral plane as Im_ref_^C^. We could then compare scores for all images that had been downloaded for a given image series and choose the image with the highest score. Images from each image series that corresponded to more medial and lateral reference images could then be identified based on their relative medio-lateral location (calculated using the ABA API database xml file corresponding to the relevant image series using the *position* and *referenceatlasindex* xml tags), since images within image series were always separated by 100, 200 or 400 μm.

The procedure for the SVM followed several stages. (a) A Gaussian filter was applied to preprocessed ISH images, with a window size of 15 and a standard deviation of 3. (b) We trained the SVM classifier with PHOW features extracted from images manually classified for 501 genes into mediolateral groups. (c) We optimized the regularization parameter of the SVM and tested performance with a further 483 genes. After training, all the remaining images were run through the SVM and assigned a score. The highest scoring image from each image series was then assigned to an Im_ref_^C^ folder for manual inspection. Any images that did not belong in Im_ref_^C^ were removed. Images that were distantly located from Im_ref_^C^ images were placed in a No-ML folder. We manually checked this folder and any images that appeared to match a reference image were moved to the appropriate folder. Records were kept of all movements and this process of checking continued until we were satisfied that images had been assigned to a reference with ∼95% accuracy.

**Registration gene images to a reference image.** Each pre-processed ISH image was Gaussian-filtered and contrast-enhanced to facilitate extraction of large anatomical features and then 1-to-1 registered to its respective reference image, also Gaussian filtered, using the MIRT toolkit (S1A Fig.). 1-to-1 registration is unlikely to be as accurate as group registration but group registration would be unfeasible, in terms of memory and time required, for the number of images involved (∼ 20,000). Images were registered using cubic β-splines with mainly default settings, with the exception of the *transformation regularization weight*, which sets a limit on the scale of the deformation. We increased this from the default value of 0.01 to 0.1 to prevent large deformations. MI was used as a similarity measure because of its invariance to differences in contrast. The output of the algorithm was a transform describing the deformation of all points.

**Apply registration transformation to expression images.** The transform, calculated using the thresholded images, was also applied to the original ISH images (for visual assessment of registration success), as well as to the expression images (for extracting pixel intensity). All images underwent the same procedure.

**Image quality check.** To assess the accuracy of registration we used several measures automatically collected from all images: an MI-related score from the registration algorithm, a cross-correlation score on the final image, and a classification score from a classifier trained on poorly registered images. The MI score of the final deformation for each image reflects its similarity to the reference image. There is a clear distinction between the distribution of scores before and after registration (S1A Fig.). To determine how well these scores represent correct alignment, we chose a random sample of 100 images and manually rated their registration accuracy, then plotted their scores against the final mutual information result. This allowed us to set a threshold so that we could flag images that were potentially poorly registered. A total of 17% of images in the central plane were flagged compared with 14% in the L1 plane.

The MI score reflects registration accuracy across the whole image and therefore could overestimate the accuracy of registration in the MEC. Therefore as a second test we used the Matlab function (*normxcorr2*) to cross-correlate the MEC-containing region of a registered image with a larger region of the respective reference image. This cross-correlation function provides both a normalized maximum fit score and the location of the maximum fit, thereby enabling us to estimate the offset between the posterior MEC border within the registered image and within the reference image. We validated scores by using the image set used for validation of MI analysis. The majority of images were given a high manual rating of 5 and had a cross-correlation offset of near zero. A total of 7% of central images were flagged as having an offset that was potentially too large.

To detect image flaws including holes in the tissue created by bubbles, and aberrant detection of the pial surface as an RNA-expressing cell, which artificially increases the mean pixel intensity of the image, we used an image classifier. We decided to use image features to capture erroneous elements in the expression images that could subsequently be detected using an SVM. Features were extracted from the region including the MEC and immediate surround. We then trained a binary SVM classifier with a radial basis function kernel on all the images that we had visually assessed as having significant errors (n=∼ 50) against high quality images (n = 300). We used the LIBSVM package in Matlab for this [99]. We used cross validation to optimize the regularization parameter, C, and the hyperparameter of the radial basis function, gamma. We then tested all images with the classifier, giving a probability estimate that each image was erroneous. We again compared the probability estimate with visual assessment of a subset of the images used previously to estimate accuracy for the MI score, and flagged images with scores greater than 0.13. The classifier distinguishes images with large registration errors and pial surface errors from high-quality images (S1A Fig.). However, this method is naive to anatomy and not particularly sensitive to minor misalignment errors as it extracts features from the entire MEC region that are scale and alignment-invariant. 29% of images were flagged based on these results. Images were assigned error statuses and defined as not meeting the quality criteria if they had poor MI scores or were flagged by at least 2 of the other error measures. Error statuses were updated for all visually assessed images. In summary, 15,447 / 20,032 (77%) genes had images meeting these quality criteria in the central plane and 12,814 (64%) had images meeting the criteria in the L1 plane (S1B Fig.).

**Extraction of pixel intensities from ABA images.** Custom python scripts were written for all analysis of 8-bit ABA expression TIFF images. Expression images were used instead of raw ISH images because stages of processing that control noise, background illumination and contrast invariance across the images have already been performed as part of development of the ABA [34, 36, 100] (see ABA Informatics Data processing white paper). In addition, pixel intensity information represents overall expression level of individual cells that have been detected as expressing the gene of interest and should not contain structural information present in brightfield images that is not gene-specific, such as densely fibrous regions,

Genes were classified as being expressed if the mean expression in either the custom-defined dorsal or ventral region was ≥ 1 (scale up to 255). All data presented are based on pixel intensity values from 8-bit grayscale expression images. Expression images have been shown with a 16-color lookup table for visualization purposes. Average images are shown either based on absolute intensity or intensity normalized by the mean of the MEC region, as indicated.

Regional gene expression was estimated by manually outlining regions using Bezier lines (ImageJ), using the selections to create a binary mask (black on white pixels) that could be imported using custom Python or Matlab scripts, then using the mask to select elements of the original expression images.

Some genes are represented more than once in the ABA dataset, either because multiple probes have been used to detect different transcripts (n = 351 / 20,334 genes), or where ISH experiments with a single probe have been replicated (n = 1,011 genes). Where multiple probes are used to target a single gene we analyze images for each probe separately, but in population analyses we report the gene once whether only 1 probe or all probes were detected. For replications we found that relative intensities across different regions were similar in each image set, but overall image intensities could vary. We therefore analyzed average images generated by obtaining the mean relative pixel intensity across regions of interest for each image series and multiplying this by the mean pixel intensity of each whole image averaged across all relevant image series.

We extracted pixel intensity information from central and adjacent lateral images, which showed highly similar patterns of gene expression. Comparisons in mean pixel intensity between corresponding MEC layers of central and adjacent lateral images showed correlations of at least 0.945, which was higher than correlations with non-corresponding layers (< 0.938). When relative mean pixel intensities were compared between corresponding layers we found correlations of at least 0.66, compared with < 0.21 for non-corresponding layers.

**Comparison of ABA and RNA-Seq expression.** To compare the results from ABA data and RNA-Seq data, we first used the Ensembl Biomart database to match Ensembl data from RNA-Seq to Entrez IDs and Gene symbols. To correlate ABA and RNA-Seq expression, we took the mean pixel intensity of the dorsal and ventral regions, averaged across the Im_ref_^C^ and Im_ref_^L1^ planes (where images were available) and compared this to the mean FPKM of RNA-Seq dorsal and ventral samples (S1A Fig.).

### Data analysis and statistics

We describe below methods used for analyses associated with each main figure. Pearson correlation coefficients and linear regression analyses were performed using the statistics linear regression package, *lm*, in *R.* We define absolute intensity as the pixel intensity measurement in 8-bit images (range 0–255), and relative intensity as a measure reflecting the ratio of pixel intensities between two or more regions, for example layers or brain regions.

**ABA regional comparisons and combinatorial analysis (Fig. 2).** The neocortical, hippocampal, caudate putamen, amygdala, piriform and MEC regions were manually outlined using the central composite reference image and Allen Mouse Reference Atlas [34, 36] as guides. Genes with a mean pixel intensity in MEC ≥ 5 were defined as being MEC-enriched if their relative intensity compared to the comparison regions was ≥ 0.8. Genes with mean pixel intensity in MEC < 5 and ≥ 2 were defined as being MEC-enriched if their relative intensity was ≥ 0.99. In both cases the mean intensity of the compared brain region also had to be < 5. The proportion of MEC-enriched genes also expressed in the other brain regions was calculated by finding genes that did not satisfy these criteria. MEC-unique genes were identified using the same thresholds applied in comparison to all regions.

We identified pairs of genes with overlapping expression by first finding all genes that are enriched in the MEC relative to at least one other brain region and pairing them with each other. We then identified those pairs for which at least one of the pair was present in all five MEC-enriched lists. Images of genes were overlaid using ImageJ and manually inspected for degree of overlap. To aid visual assessment, genes with MEC expression < 5 were only included if expression was restricted to a particular subregion, as uniform expression at this intensity appears to most likely reflect non-specific ISH staining or uneven illumination across the tissue.

**Detection of borders (Fig. 3).** To identify genes defining the dorsal and ventral borders of MEC, we outlined regions dorsal and ventral to the approximate location of the borders using the central reference image (Fig. 3A, 3F). All gene images meeting the quality criteria in the re-registered data set with mean pixel intensity < 15 in one region and with a differential pixel intensity of > 15 between the regions were selected for manual validation. We supplemented this search with an ABA differential expression search [34, 36] (Target structure: Parasubiculum, Contrast structure: Medial Entorhinal Cortex), with an expression threshold of 3.5, which identified an additional 8 genes that demarcated the dorsal border of the MEC.

**Identification of layer-specific and differentially expressed (DE) genes (Fig. 4).** Genes were defined as layer-specific if they show no consistent expression in other MEC layers or differentially expressed (DE) if they show substantially higher expression in at least one layer than in another. Exact criteria for identification of layer-specific and DE genes are as follows.

(1) Comparison of laminar regions within the re-registered ABA dataset. Layers were defined using the composite central and adjacent lateral reference images (S4A Fig.). We primarily used data from the central reference image, only using information from the lateral plane when no central image was available. Since layers possess multiple types of cells that may themselves differentially express genes, we compared both average expression across layers, and the distribution of high-intensity expression. For each image a high-intensity pixel was defined as having intensity ≥ mean + 2 x S.D of all pixels in the MEC. The absolute pixel intensity (Lx_abs =_ Lx_rel_ x 3 x MEC_mean_, where x refers to layer) and proportion of high-intensity pixels were then calculated for each of the layers II, III and V/VI. Relative laminar mean intensity (Lx_rel_) and relative proportion of high-intensity pixels (Lx_prop_) were calculated by dividing layer measures by their sum. We also identified the first (Lmax = maximum of (LII_rel_ + LII_prop_, LIII_rel_ + LIII_prop_, LV_rel_ + LV_prop_)) and second highest expressing layers (Lmid (not Lmin or Lmax)). We then calculated an absolute mean pixel intensity difference between these layers (Lmax_diff_ = ((Lmax_rel_-Lmid_rel_)^2^ x Lmax_abs_ / 255) x 30) and the joint expression of the two highest expressing layers (Lmax_absjoint_ = ((Lmax_rel_ + Lmid_rel−_Lmin_rel_)^2^ x Lmax_abs_ / 255) x 30). These values acted to penalize low absolute pixel intensities. We did not initially distinguish layers V and VI as the border between these layers is not clearly defined in the sagittal plane. Only genes with a mean overall pixel intensity ≥ 1 and mean pixel intensity ≥ 2 in at least one layer were evaluated.

To quantify laminar differences in expression we calculated patterning scores (PS) as follows:

1. We assigned weights to the measures relative intensity (w_rel_ = 0.4), relative proportion of high-intensity pixels (w_prop_ = 0.5) and absolute pixel intensity (w_abs_ = 1- (w_rel_ + w_prop_)). If the image had no high-intensity pixels, w_rel_ = 0.9.
2. We calculated a PS for single layer enrichment: PS_single_ = w_rel_ x Lmax_rel_ + w_prop_ x Lmax_prop_ + w_abs_ x min([Lmax_abs_,1])
3. We calculated a PS for joint layer enrichment: PS_joint_ = w_mean_ x (Lmax_rel_ + Lmid_rel_) + w_prop_ x (Lmax_prop_ + Lmid_prop_) + w_abs_ x min([Lmax_absjoint_,1])

Genes with PS_single_ ≥ 0.65 or PS_joint_ ≥ 0.88 (n = 1,314) were marked as candidates for DE genes, including the subset of layer-specific genes.

(2) Cross correlation of genes within the registered data set. We also used the re-registered data set to find genes with similar patterns of intensity to genes identified through differential laminar expression, but that may have been missed in the previous analysis due to their occupation of very small areas. Using a SciPy cross-correlation function (Alistair Muldal:https://github.com/oleg-alexandrov/projects/blob/master/fft_match/norm_xcorr.py), we used *Nxph4* as the seed gene to find other layer VI genes and *Mrg1* as the seed to find other potential island genes. To identify genes only expressed in the narrow VI layer, we compared a small dorsal region and searched only images with mean pixel intensity ≥ 1 and < 5 that had a sum of squares difference (SSD) of zero with the target gene. We checked images for the 30 genes with the highest cross-correlation. For island genes, we searched using a small dorsal region including layer II and checked the top 50 genes with mean intensity ≥ 1 and SSD = 0.

(3) Identification of genes through ABA search tools. Since our aim was to make this resource as comprehensive as possible, we extended our search beyond our re-registered data set to make use of ABA differential expression tools and knowledge of cortex-enriched genes [34, 101]. We initially identified strongly differentially expressed ‘seed’ genes through manual exploration using the ABA differential expression tool, Fine structure Annotation and Anatomical Gene Expression Atlas (AGEA; http://mouse.brain-map.org/agea) (total checked = 922). Taking at least 8 genes with the strongest expression for each layer, we used the ABA NeuroBlast tool to identify all other genes with an expression correlation of at least 0.5 with any one of these ‘seed’ genes in the retrohippocampal (RHP) region. This provided us with over 4,000 potential DE genes, but no clear indication of their laminar expression profile. We visually assessed all those that did not have an image meeting the quality criteria in our re-registered ABA data set (n = 959). For the identification of layer-specific genes we also scanned all those that our analysis suggested had borderline differential expression (n = 163) or that had a NeuroBlast expression correlation greater than 0.7 (n = 1,288).

Given the potential for images to be poorly registered in both ABA and our re-registered data set, we also visually inspected differentially expressed neocortical genes (n = 302) using lists provided by [101](http://www.nature.com/nrn/journal/v8/n6/suppinfo/nrn2151.html) and those annotated as having a ‘high’ specificity score in the Somatosensory cortex Annotation on the ABA website (http://help.brain-map.org/download/attachments/2818169/SomatosensoryAnnotation.xls?version=1&modificationDate=1319171046372) [34, 36] to determine whether they also showed laminar specificity in MEC.

(4) Visual validation. To minimize the number of false positives in the data, all candidate layer-specific and DE genes were validated through visual inspection. Layer-specific genes were confirmed as showing consistent expression in a single layer using the original ISH image and, where possible, images in more lateral and medial planes. Genes with DE expression had to show consistently higher expression in at least one layer than in another. For ABA re-registered data, 703 / 1314 candidates could be added to the set of DE genes. An additional 4 genes with layer-specific expression were identified using cross-correlation and an additional 36 DE including 10 layer-specific genes using the cortex-enriched lists referred to above. The NeuroBlast data identified an additional 121 DE genes, 13 of which were layer-specific. During manual validation of DE genes, we also recorded particular patterns of gene expression, including island or inter-island expression and specific laminar expression within the deep layers.

To generate final lists of layer-specific genes, we included all genes visually validated as layer-specific, independent of PS score. For DE genes, we included all layer-specific genes, all genes in the re-registered ABA data set that had a single layer PS ≥ 0.65 or joint PS ≥ 0.88 and that were visually assessed as strongly differentially expressed, and all genes acquired using ABA tools and cortex layer-enriched lists that we validated as showing differential expression. See S4B Fig. Layer-specific and DE genes showed consistent expression patterns across mediolateral sections (S6 Fig.).

**Analysis of laminar similarities and differences between MEC and neocortex in ABA data (Fig. 5).** Taking the neocortical region used in our analysis of regional expression, we associated all pixel intensities for each gene image with a normalized location relative to the corpus callosum (or subicular border for MEC) and the nearest point along the pial surface. For all MEC layer-specific genes (S4C Fig.), we calculated mean pixel intensities at different normalized locations throughout the three cortical regions (Fig. 5B). We plotted histograms by binning the distances into 20 regions. Statistically significant differences in the laminar gene list expression patterns were detected using Mixed Model Analysis in SPSS (v21) with an unstructured covariance matrix. Fixed effects were the list of genes, location and their interaction. Random effects were the list of genes and location with image series as subject.

To test whether deep and superficial layer-specific expression patterns correspond between MEC and neocortex, we used genes previously identified as having ‘high’ specificity in SS cortical layers [34] (S5E Fig.) to divide the cortical regions into deep (layers V/VI) and superficial (II-IV) regions (cf. [101]). We also used these genes to estimate an approximate border between visual and SS cortex. For each gene with mean pixel intensity ≥ 2, we calculated the ratio of pixel intensity in the deep region to the superficial region. If gene expression in MEC and visual or SS cortex corresponds, we would expect this ratio to be 1 for MEC deep layer-specific genes and 0 for MEC superficial layer genes. To test this prediction, we subtracted the ratio for each gene from the expected value and performed a MANOVA using SPSS (v21) with type of MEC specificity as the between-subjects variable and the visual and SS results as dependent variables. Post-hoc tests were performed with Tukey’s HSD. The percentage of genes that are enriched in superficial or deep regions was calculated by including genes with a deep: superficial ratio of less than 0.4 or greater than 0.6 (50% difference). Genes with mean intensity < 2 in the neocortical region were not included in the analysis.

To calculate the correlations between cortical layers across all ABA genes, we used the estimated laminar boundaries described previously to generate binary array masks corresponding to each layer. Pixel intensities were averaged across all pixels within laminar boundaries then Pearson correlation coefficients calculated.

**Functional analysis for genes with laminar organization (Fig. 6).** We used the GOElite gene ontology tool [102] with recent versions of the GO OBO database and gene annotation file for *mus musculus* (24/1/2014) and Kyoto Encyclopaedia of Genes and Genomes (KEGG) database [63] to extract all enriched terms. For the reference set for DE genes, all DE genes and genes with weak differential or uniform expression and threshold mean intensity ≥ 2 in MEC (n = 9,057 Ensembl identifiers) were included. This ensured we were not simply selecting for brain-enriched genes but for genes differentially expressed within the MEC. Terms were defined as enriched if associated with a *p* value < 0.05 after a one-sided Fisher overrepresentation test followed by Benjamini-Hochberg false discovery rate adjustment for multiple tests.

To reduce redundancy and identify clusters of meaningful GO and KEGG terms we calculated a kappa similarity measure (described in [103]) to identify terms sharing a higher proportion of genes than chance. We then used hierarchical clustering (adapted from code written by Nathan Salomonis: http://code.activestate.com/recipes/578175-hierarchical-clustering-heatmap-python/) on the kappa similarity matrix to cluster terms with at least 5 genes and fewer than 100 genes into groups. Within each cluster we then extracted all kappa similarity scores. Only terms with less than modest overlap (kappa < 0.7) with a more significant term and fewer than 100 genes were presented in the summary figure (Fig. 6A). Clusters were labelled using their most significant term. To establish the significance of the over or under representation of layer-specific genes in the selected lists we used *R* implementation of a 2-way Exact Fisher test (*fisher.test*) followed by multiple corrections analysis (*p.adjust.M*). In heatplots, data are shown for images in the plane corresponding to the central reference image, where available, or for the adjacent lateral plane.

**Investigating dorsoventral differences in gene expression (Fig. 7).** For analysis of RNA-Seq data Cuffdiff 2 [58] was used to identify all differentially expressed genes with an FPKM of at least 1 and difference in expression of at least 20% (log_2_(1.2) = 0.2630). Differences in the direction of differential expression between genes with different layer-specific expression (Fig. 4) were detected using ANOVA followed by post-hoc Tukey’s HSD tests, performed in R.

For analysis of ABA images dorsal and ventral regions were manually outlined using the two primary custom reference atlases. Average pixel intensities within each region and across central and the adjacent lateral sections containing MEC were calculated for each ABA image series. Only ABA images with a threshold mean intensity ≥ 2 in the dorsal or ventral region were included in the differential expression analysis. We used a threshold log fold enrichment of 0.2630 (increase of 20%) to define differential dorsoventral expression, the same value used for RNA-Seq.

For comparison between Cuffdiff 2 analysis and ABA analysis, Cuffdiff 2 results were matched to ABA Entrez values or gene official symbols using the Ensembl Biomart tool. Two comparisons were made: one specific to ABA layer-specific genes, and the other only including genes determined to be significantly differentially expressed according to RNA-Seq data.

**Functional analysis for dorsoventral genes (Fig. 8).** A similar method was used to that described above for laminar genes. For the reference set we included all genes with an FPKM ≥ 1 in MEC that had a sufficient number of alignments in a locus (measured using Cuffdiff 2) to be tested for differential expression (Ensembl identifiers = 13,954).

To estimate the presence of gradients in MEC in re-registered ABA data, we divided the MEC region in both the central and adjacent lateral planes into 5 subregions along the dorsoventral axis. For each layer and subregion, we calculated the mean pixel intensity and used these values to calculate linear regression gradients (SciPy *stats.linregress)* for each layer and across the whole MEC. For heatmaps, genes were sorted according to this gradient and color-coded according to whether their dorsoventral difference was sufficiently large for them to be defined as D>V or V>D-expressed in the analysis performed in Fig. 6.

**Disease analysis of layer-patterned genes (Fig. 9).** Genes involved in pathways for Alzheimer’s disease, Huntington’s disease and Parkinson’s disease were acquired from the KEGG pathway database [63]. This tool contains manually drawn pathways of molecules known to be perturbed, either through environment or genes, in certain diseases, as well as molecules that are therapeutic markers or diagnostic markers. Autism-related genes include mouse genes for which the human homolog has a score of 1–4 (high-low evidence) or syndromic in the SFARI AutDB database (n = 238 (Ensembl)). Schizophrenia genes include the results of a computational analysis of meta-analysis results from [64, 104] (n = 175 (Ensembl)). Epilepsy genes include those associated with any form of epilepsy in the Disease database (n = 90 (Ensembl)) [67]. Genes that have been causally associated with early or late-onset AD were obtained from key reviews and meta-analyses in the AD literature [69, 72, 105].

To generate lists of layer-enriched genes we took all DE genes and identified those visually validated as being enriched in particular layers. We computed the significance of over or underrepresentation of disease genes amongst layer-enriched genes using Fisher’s Exact Test in *R*, as described for gene ontologies in Fig. 6. To determine the associated ontology terms of all AD pathway genes with at least moderate layer enrichment we used a PS_single_ threshold of 0.60.

## Supporting Information

S1 Fig Generation of high-resolution data set from ABA ISH data. Related to Fig. 1. (A) Pipeline for image processing stages involved in extracting pixel intensity information from images in the Allen Brain Atlas (ABA): (1) Raw ISH and corresponding expression images were downloaded from the ABA using the API http://www.brain-map.org/api/index.html (see Materials and Methods). (2) Raw ISH images were pre-processed to improve registration performance. This involved scaling (to approx. 10µm per pixel), background subtraction, thresholding, median filtering and Gaussian blurring. The cerebellar region was removed using object segmentation. (3) Generation of the reference images (Im_ref_). For each ML region 15–20 images were chosen (central images shown here). These images were manually aligned using rigid registration (ImageJ) to a template image (red dotted line), then registered using non-linear deformation. Im_ref_ was defined as the median of the resulting images (lower). (4) Images were classified using an SVM with a linear kernel into groups based on their medio-lateral extent. (5) The registration procedure. For each gene a registration transformation is calculated. The effect of registration is shown for 2 example ISH images before and after 1-to-1 registration using MIRT to Im_ref_^C^, the median image produced in (4). (6) The registration transformation generated by MIRT is applied to the corresponding expression image. (7) Image quality was assessed using several metrics. 1. The mutual information-related (MI) score reached during registration reflects the similarity between the reference image and registered image. Images with poor MI scores were flagged (red region) and only included in subsequent analyses after visual checks. 2. An SVM was trained on poor images and high quality images. The resulting model was then applied to unchecked images and a probability of being erroneous assigned. Those with a probability of being erroneous of greater than 0.13 were also flagged (red) and only included after checking. (8) Pixel intensity data were extracted from images using custom-made python scripts. (9) Data from ABA images were compared to RNA-Seq analysis data. Images show examples of the brain slices from which dorsal and ventral regions were removed and used for RNA-Seq analysis. Arrows indicate the cuts marking the border between the dorsal and ventral region. (B) Table summarizing the number of images (unique genes) satisfying the quality criteria (Materials and Methods) in the two planes used for analysis: C and L1.
(TIFF)

S2 Fig Gene expression differences across brain regions. Related to Fig. 2. (A) Scatter plot of absolute mean pixel intensity (mINT) in neocortex as a function of mINT in MEC across all genes in the ABA re-registered data set (red: slope = 0.918, *r* = 0.958, *p* < 2.2 × 10^-16^). (B) Results of linear regression used to determine the relationship between expression in MEC and neocortex (red), hippocampus (green), amygdala (magenta), piriform cortex (blue) and caudate putamen (cyan). Shaded regions indicate the areas in which 95% of data points fall. (C) Heat plots show the distribution of relative mean pixel intensities in MEC compared with other brain regions (mINT_norm_^MEC^). A result of 1 indicates that expression is unique to MEC, 0.5 indicates equal expression and 0 indicates expression only in the other brain region. Only genes with mINT^MEC^ ≥ 5 are shown. MEC-enriched genes are defined as those where mINT_norm_^MEC^ ≥ 0.8. (D) Plot shows the effect of fold-change threshold on the number of genes detected as being enriched in MEC compared with each other region. At thresholds of 3.5, 4 and 4.5 the numbers of genes that distinguish MEC from each area are: Neo [149, 118, 96], Hip [73, 54, 43], Amygdala [354, 318, 288], Caudate [1253, 1162, 1057], Piriform [98, 93, 81]. (E) Expression images for examples of genes with different expression patterns in all regions except MEC. Within MEC this expression is overlapping (upper), partially overlapping (mid) or restricted to different layers (lower). Overlay images were created by taking 8-bit grayscale original images for each, performing contrast enhancement (1% saturated pixels) then smoothing for viewing purposes using ImageJ functions. Images were merged using the ‘Color: Merge’ function and brightness/contrast adjusted where appropriate. White boxed outline regions in MEC shown at higher magnification. (F) Bar chart showing the relative numbers of pairs of genes, out of all pairs identified as having potentially overlapping gene expression in MEC but not other regions, manually sorted according to particular pattern of overlap or with additional non-specific expression in other regions.
(TIFF)

S3 Fig Gene expression-based border demarcations throughout the medio-lateral extent. Related to Fig. 3. (A-D) Example cropped ISH images downloaded from the ABA API (see Materials and Methods) are shown for genes with expression patterns that distinguish the (A-B) dorsal and (C-D) ventral borders of the MEC. Images corresponding to the central reference image are shown adjacent to more lateral and more medial images. (A) Dorsal-MEC^+ve^, (B) Dorsal-Para, (C) Ventral-MEC^+ve^ and (D) Ventral-MEC^-ve^.
(TIFF)

S4 Fig Layer-specific gene expression in MEC. Related to Fig. 4. (A) Schematic shows the central reference image overlaid with ROIs corresponding to layers II (red), III (green) and V/VI (blue) of MEC. (B) Table summarizes the number of layer-specific and differentially expressed (DE) genes detected. (C-G) Example cropped ISH images downloaded from the ABA API (see Materials and Methods) are shown adjacent to images of the mean expression pattern of genes with images in the central plane of the re-registered ABA data set. Patterns include: (C) Layer-specific gene expression in each of the three major laminar regions. (D) Deep-layer specific patterns including layer Va, layer Vb and layer VI. (E) Enriched expression in particular deep layers or combinations of deep layers. (F-G) Sub-laminar expression patterns within MEC layer II, including island and inter-island patterns. (F) All DE genes with this pattern. (G) Layer II-specific genes.
(TIFF)

S5 Fig Neocortical expression patterns of MEC layer-specific genes. Related to Fig. 5. (A) Normalized intensity of MEC layer-specific genes plotted as a function of distance from the inner white matter border in MEC, visual or SS cortex. There is a main fixed effect of MEC layer-specific group on normalized neocortical expression (Mixed Model Analysis, *F* = 28.8, *p* < 0.001). For each gene, pixel intensities were normalized to the sum across the whole region. Error bars represent standard error of the mean. (B) Plots show the distribution of absolute pixel intensities for individual MEC layer-specific genes in each region (LII: upper, red, LIII: mid, green, LV/VI: lower, blue). (C) Table showing summary statistics for differences in neocortical expression pattern. (D) Examples of section-wide images downloaded from the ABA API (see Materials and Methods) of genes with layer-specific expression patterns. (E) As Fig. 5C and (A), for genes enriched in SS layer II/III, IV, V, VIa and VIb.
(TIFF)

S6 Fig Consistent layer-specific gene expression throughout the medio-lateral extent. Related to Figs. 4 and 5. (A-B) Images show the mean expression patterns of genes in the re-registered ABA data set that show (A) layer-specific or (B) layer-enriched gene expression in each of the three major laminar regions. Images are shown for the central (C), adjacent lateral (L1) and adjacent medial (M1) sections. (C) Example ISH images at different mediolateral extents are shown for layer-specific genes in layers II, III and V/VI.
(TIFF)

S7 Fig Dorsoventral gradients in MEC. Related to Fig. 7. (A) Histogram shows the distribution of differences in dorsoventral expression (log_2_ (mINT Ventral / mINT Dorsal)) for all genes (black) with mINT ≥ 2 in the ABA re-registered data set. Colored boxes indicate the scores corresponding to genes classified with differential expression: D>V (purple) or V>D (orange). (B) The log fold change in ABA mINT between ventral and dorsal regions is plotted as a function of log fold change in RNA-Seq mean FPKM for genes found with Cuffdiff 2 to have statistically significant (FDR < 0.05) dorsoventral differences in RNA-Seq mean FPKM. Colors represent number of genes. The linear regression line is indicated in magenta. (C) Table shows dorsoventral differences in ABA and RNA-Seq expression for all layer-specific genes identified as D>V or V>D according to Cuffdiff 2 analysis.
(TIFF)

S1 Dataset ABA and RNASeq results in Fig. 1. (XLS)

S2 Dataset Brain region data for Fig. 2. (XLS)

S3 Dataset Border genes for Fig. 3. (XLS)

S4 Dataset Differentially expressed and layer-specific genes in Fig. 4. (XLS)

S5 Dataset Gene expression in neocortical layers in Fig. 5. (XLS)

S6 Dataset Gene ontology terms for differentially expressed genes in Fig. 6. (XLS)

S7 Dataset Genes with dorsoventral gradients in Fig. 7. (XLS)

S8 Dataset Gene ontology terms for genes with dorsoventral gradients in Fig. 8. (XLS)

S9 Dataset Gene lists for disease-associated genes in Fig. 9. (XLS)

S10 Dataset Combinatorial gene expression in MEC for S2 Fig. (XLS)
