# Supplementary figures and images for: Laminar and Dorsoventral Molecular Organization of the Medial Entorhinal Cortex Revealed by Large-scale Anatomical Analysis of Gene Expression
Source: PLoS Comput Biol. 2015 Jan 23;11(1):e1004032. doi: 10.1371/journal.pcbi.1004032 (PMC4304787; doi:10.1371/journal.pcbi.1004032)

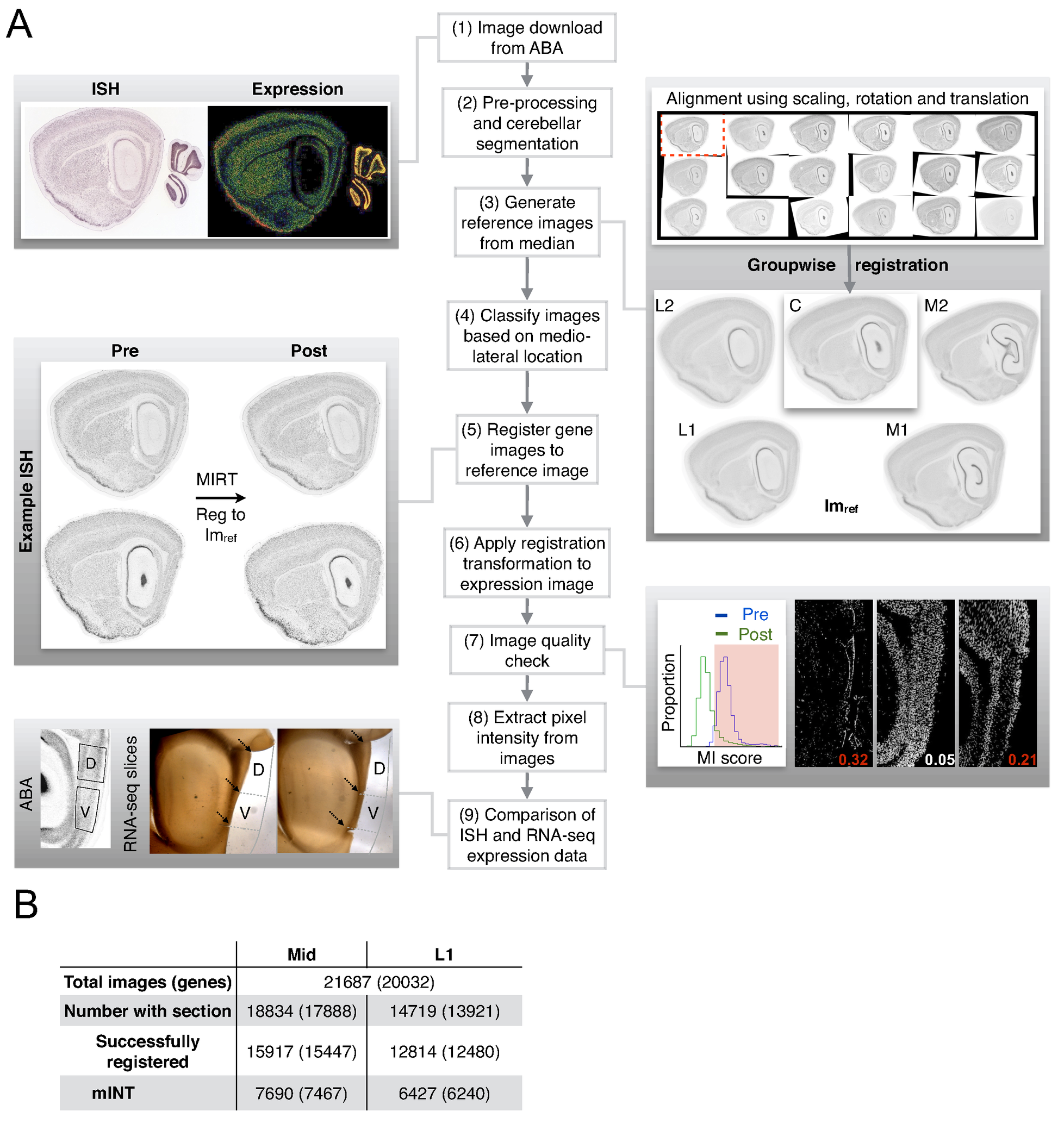

Supplement: S1 Fig — Related to Fig. 1. (A) Pipeline for image processing stages involved in extracting pixel intensity information from images in the Allen Brain Atlas (ABA): (1) Raw ISH and corresponding expression images were downloaded from the ABA using the API http://www.brain-map.org/api/index.html (see Materials and Methods). (2) Raw ISH images were pre-processed to improve registration performance. This involved scaling (to approx. 10µm per pixel), background subtraction, thresholding, median filtering and Gaussian blurring. The cerebellar region was removed using object segmentation. (3) Generation of the reference images (Imref). For each ML region 15–20 images were chosen (central images shown here). These images were manually aligned using rigid registration (ImageJ) to a template image (red dotted line), then registered using non-linear deformation. Imref was defined as the median of the resulting images (lower). (4) Images were classified using an SVM with a linear kernel into groups based on their medio-lateral extent. (5) The registration procedure. For each gene a registration transformation is calculated. The effect of registration is shown for 2 example ISH images before and after 1-to-1 registration using MIRT to Imref C, the median image produced in (4). (6) The registration transformation generated by MIRT is applied to the corresponding expression image. (7) Image quality was assessed using several metrics. 1. The mutual information-related (MI) score reached during registration reflects the similarity between the reference image and registered image. Images with poor MI scores were flagged (red region) and only included in subsequent analyses after visual checks. 2. An SVM was trained on poor images and high quality images. The resulting model was then applied to unchecked images and a probability of being erroneous assigned. Those with a probability of being erroneous of greater than 0.13 were also flagged (red) and only included after checking. (8) Pixel inten [file pcbi.1004032.s001.tiff]

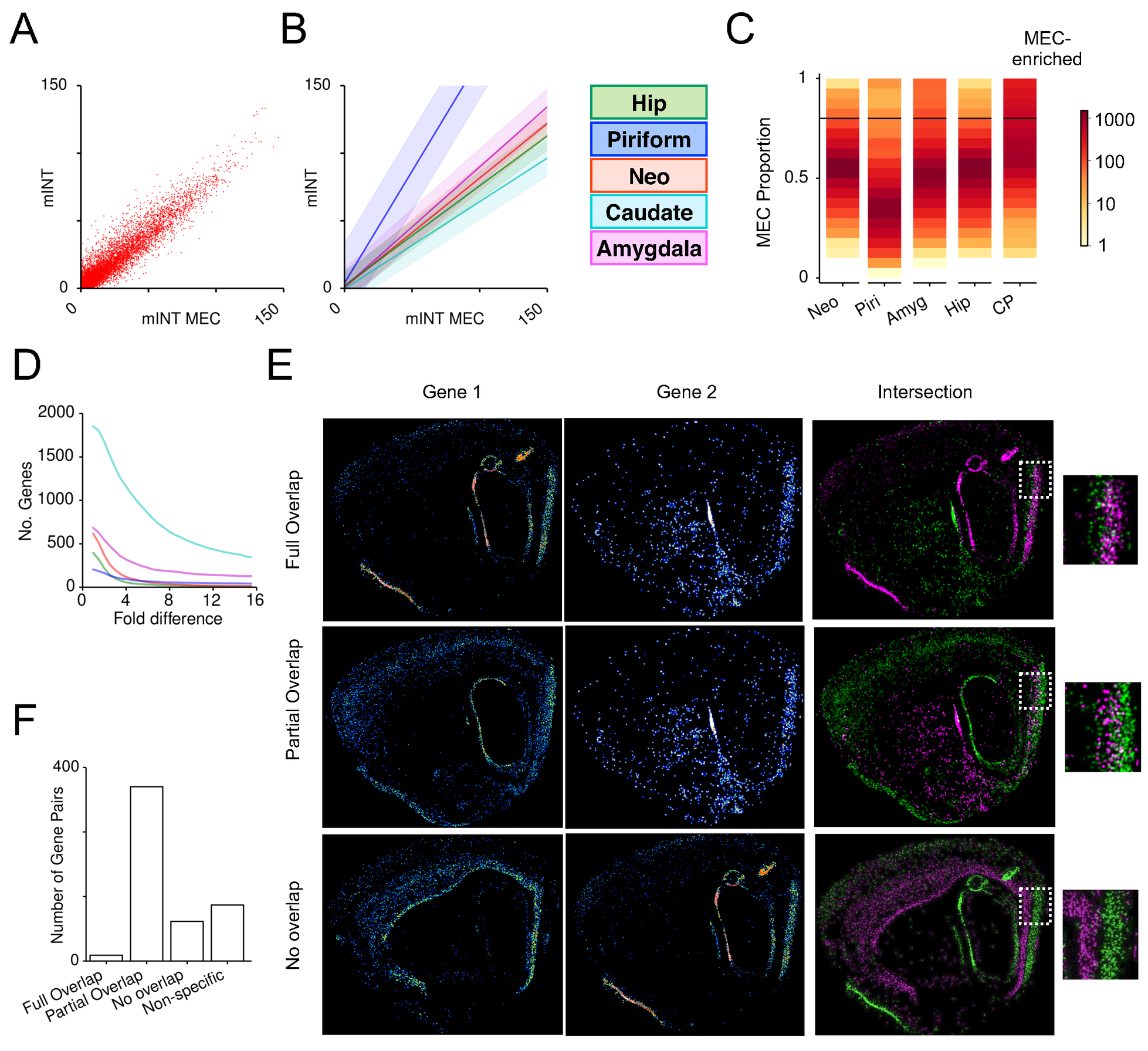

Supplement: S2 Fig — Related to Fig. 2. (A) Scatter plot of absolute mean pixel intensity (mINT) in neocortex as a function of mINT in MEC across all genes in the ABA re-registered data set (red: slope = 0.918, r = 0.958, p < 2.2 × 10^-16^). (B) Results of linear regression used to determine the relationship between expression in MEC and neocortex (red), hippocampus (green), amygdala (magenta), piriform cortex (blue) and caudate putamen (cyan). Shaded regions indicate the areas in which 95% of data points fall. (C) Heat plots show the distribution of relative mean pixel intensities in MEC compared with other brain regions (mINTnorm MEC). A result of 1 indicates that expression is unique to MEC, 0.5 indicates equal expression and 0 indicates expression only in the other brain region. Only genes with mINTMEC ≥ 5 are shown. MEC-enriched genes are defined as those where mINTnorm MEC ≥ 0.8. (D) Plot shows the effect of fold-change threshold on the number of genes detected as being enriched in MEC compared with each other region. At thresholds of 3.5, 4 and 4.5 the numbers of genes that distinguish MEC from each area are: Neo [149, 118, 96], Hip [73, 54, 43], Amygdala [354, 318, 288], Caudate [1253, 1162, 1057], Piriform [98, 93, 81]. (E) Expression images for examples of genes with different expression patterns in all regions except MEC. Within MEC this expression is overlapping (upper), partially overlapping (mid) or restricted to different layers (lower). Overlay images were created by taking 8-bit grayscale original images for each, performing contrast enhancement (1% saturated pixels) then smoothing for viewing purposes using ImageJ functions. Images were merged using the ‘Color: Merge’ function and brightness/contrast adjusted where appropriate. White boxed outline regions in MEC shown at higher magnification. (F) Bar chart showing the relative numbers of pairs of genes, out of all pairs identified as having potentially overlapping gene expression in MEC but not other regions, manually so [file pcbi.1004032.s002.tiff]

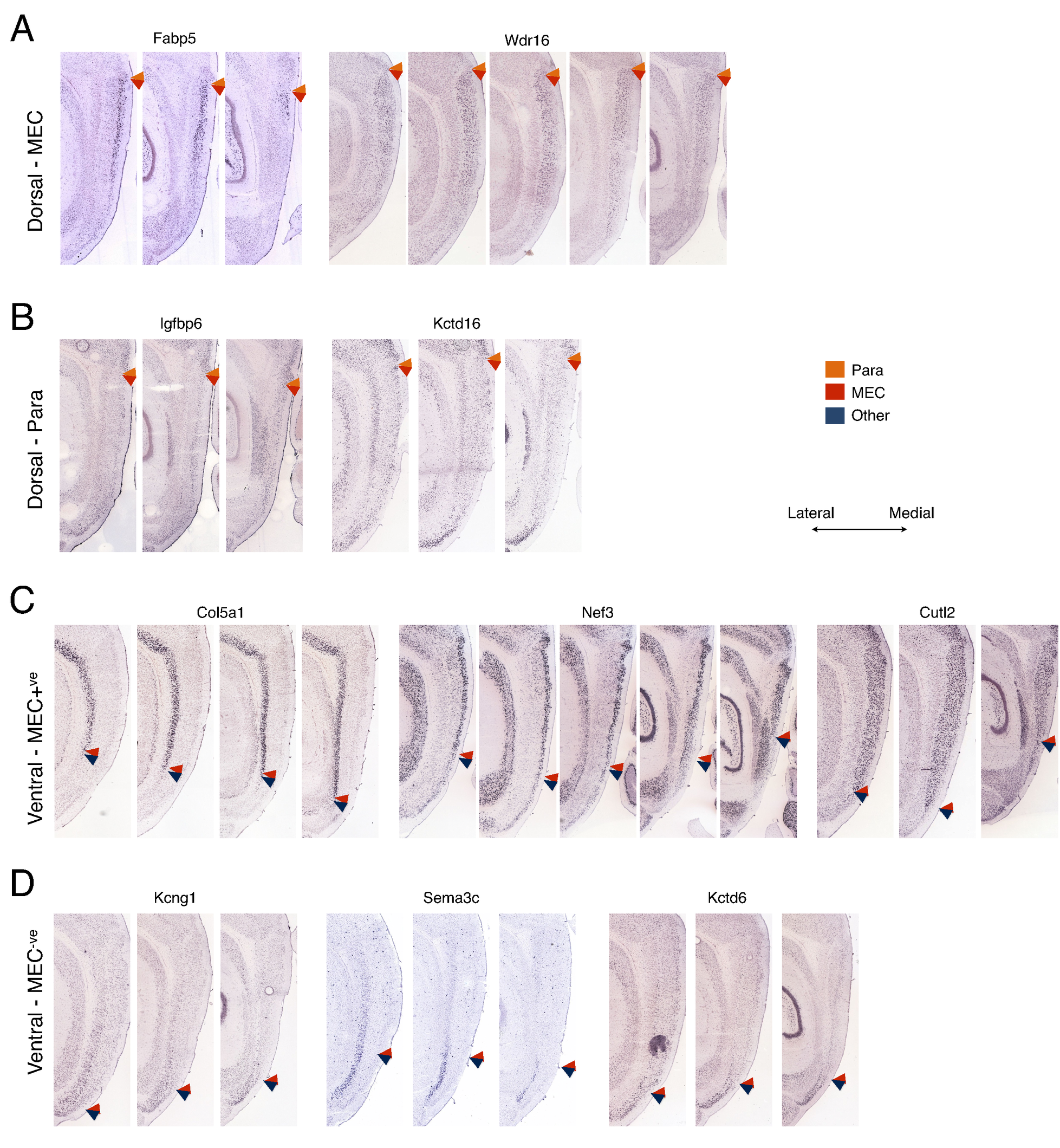

Supplement: S3 Fig — Related to Fig. 3. (A-D) Example cropped ISH images downloaded from the ABA API (see Materials and Methods) are shown for genes with expression patterns that distinguish the (A-B) dorsal and (C-D) ventral borders of the MEC. Images corresponding to the central reference image are shown adjacent to more lateral and more medial images. (A) Dorsal-MEC+ve, (B) Dorsal-Para, (C) Ventral-MEC+ve and (D) Ventral-MEC-ve. (TIFF) [file pcbi.1004032.s003.tiff]

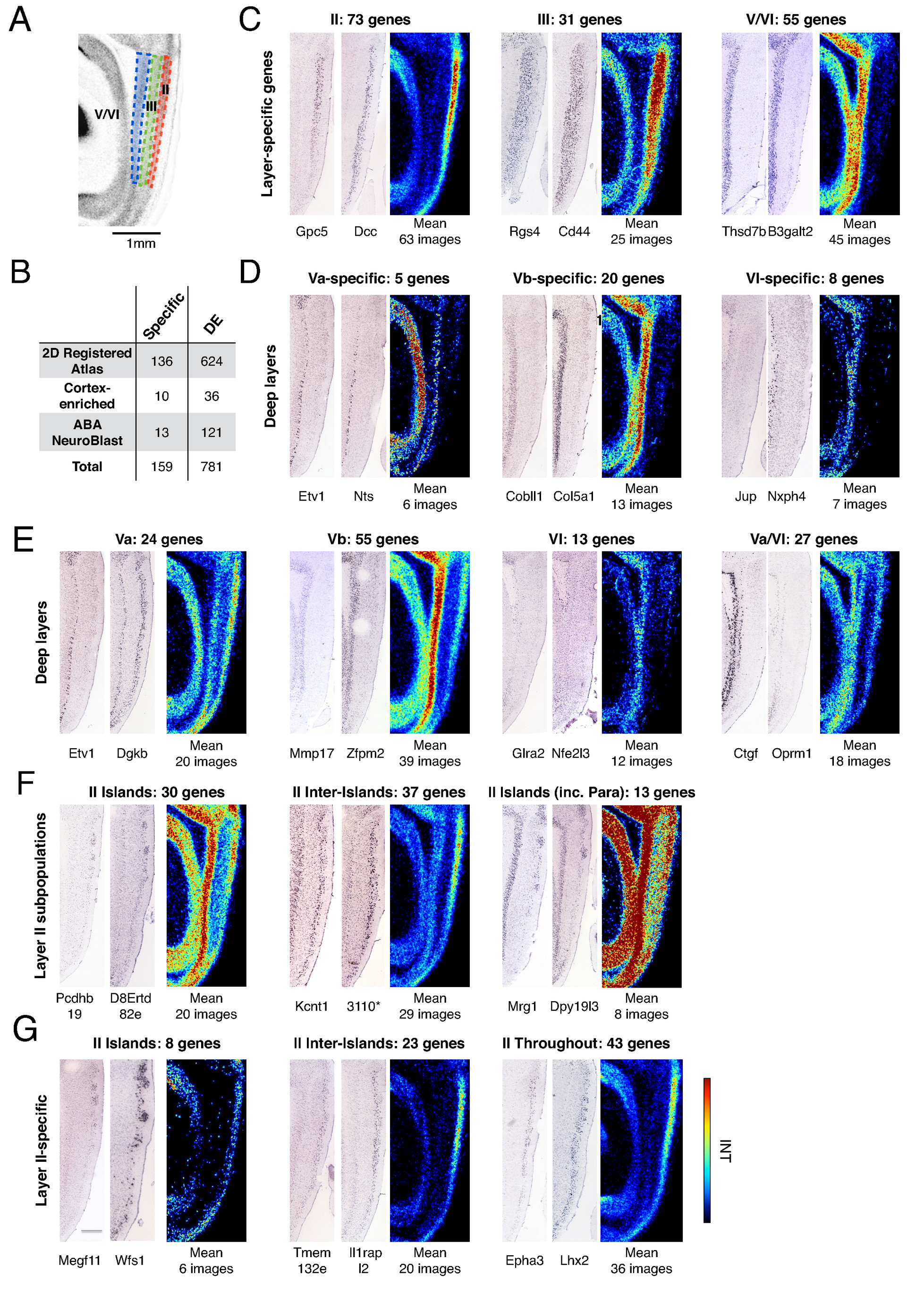

Supplement: S4 Fig — Related to Fig. 4. (A) Schematic shows the central reference image overlaid with ROIs corresponding to layers II (red), III (green) and V/VI (blue) of MEC. (B) Table summarizes the number of layer-specific and differentially expressed (DE) genes detected. (C-G) Example cropped ISH images downloaded from the ABA API (see Materials and Methods) are shown adjacent to images of the mean expression pattern of genes with images in the central plane of the re-registered ABA data set. Patterns include: (C) Layer-specific gene expression in each of the three major laminar regions. (D) Deep-layer specific patterns including layer Va, layer Vb and layer VI. (E) Enriched expression in particular deep layers or combinations of deep layers. (F-G) Sub-laminar expression patterns within MEC layer II, including island and inter-island patterns. (F) All DE genes with this pattern. (G) Layer II-specific genes. (TIFF) [file pcbi.1004032.s004.tiff]

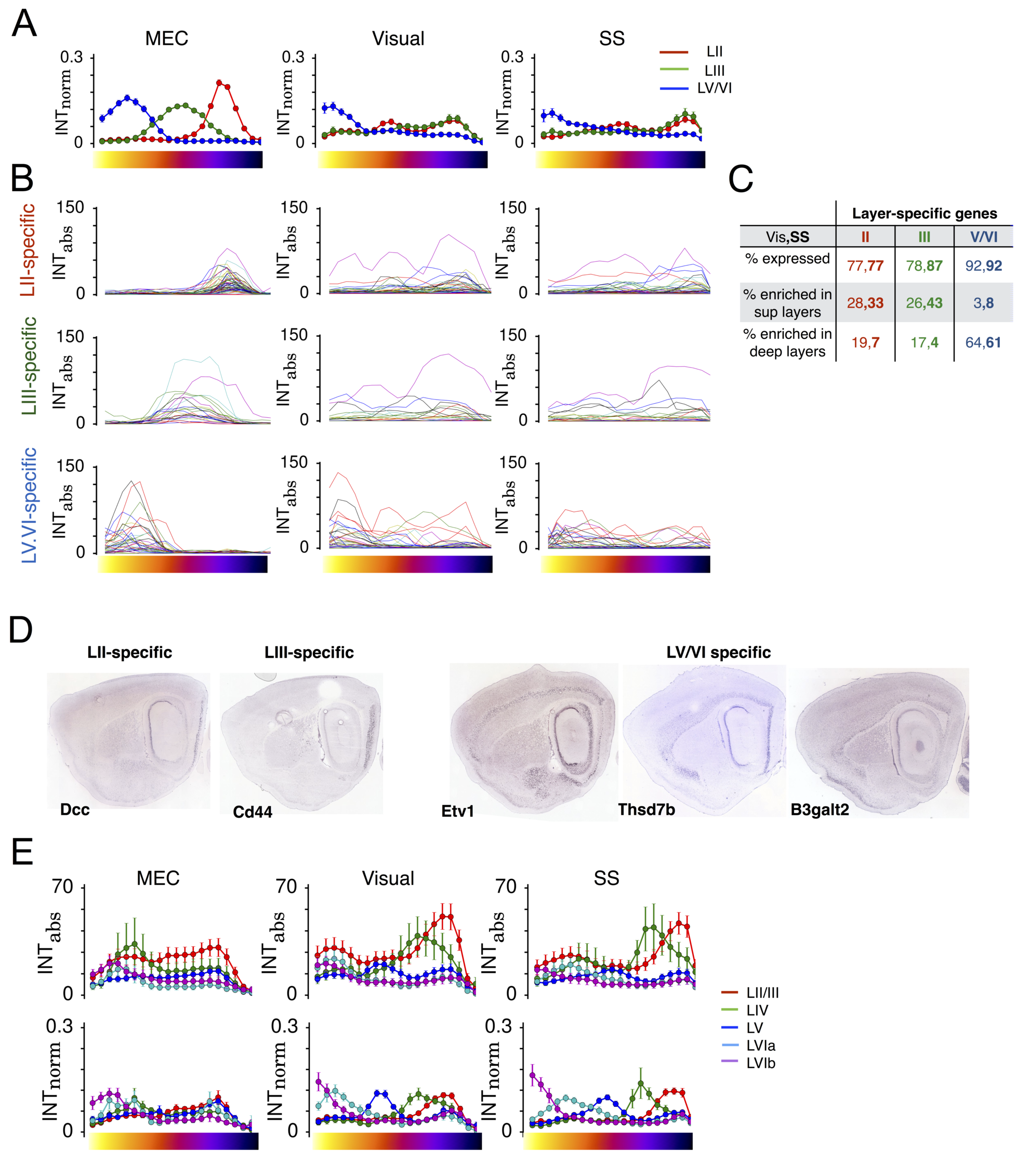

Supplement: S5 Fig — Related to Fig. 5. (A) Normalized intensity of MEC layer-specific genes plotted as a function of distance from the inner white matter border in MEC, visual or SS cortex. There is a main fixed effect of MEC layer-specific group on normalized neocortical expression (Mixed Model Analysis, F = 28.8, p < 0.001). For each gene, pixel intensities were normalized to the sum across the whole region. Error bars represent standard error of the mean. (B) Plots show the distribution of absolute pixel intensities for individual MEC layer-specific genes in each region (LII: upper, red, LIII: mid, green, LV/VI: lower, blue). (C) Table showing summary statistics for differences in neocortical expression pattern. (D) Examples of section-wide images downloaded from the ABA API (see Materials and Methods) of genes with layer-specific expression patterns. (E) As Fig. 5C and (A), for genes enriched in SS layer II/III, IV, V, VIa and VIb. (TIFF) [file pcbi.1004032.s005.tiff]

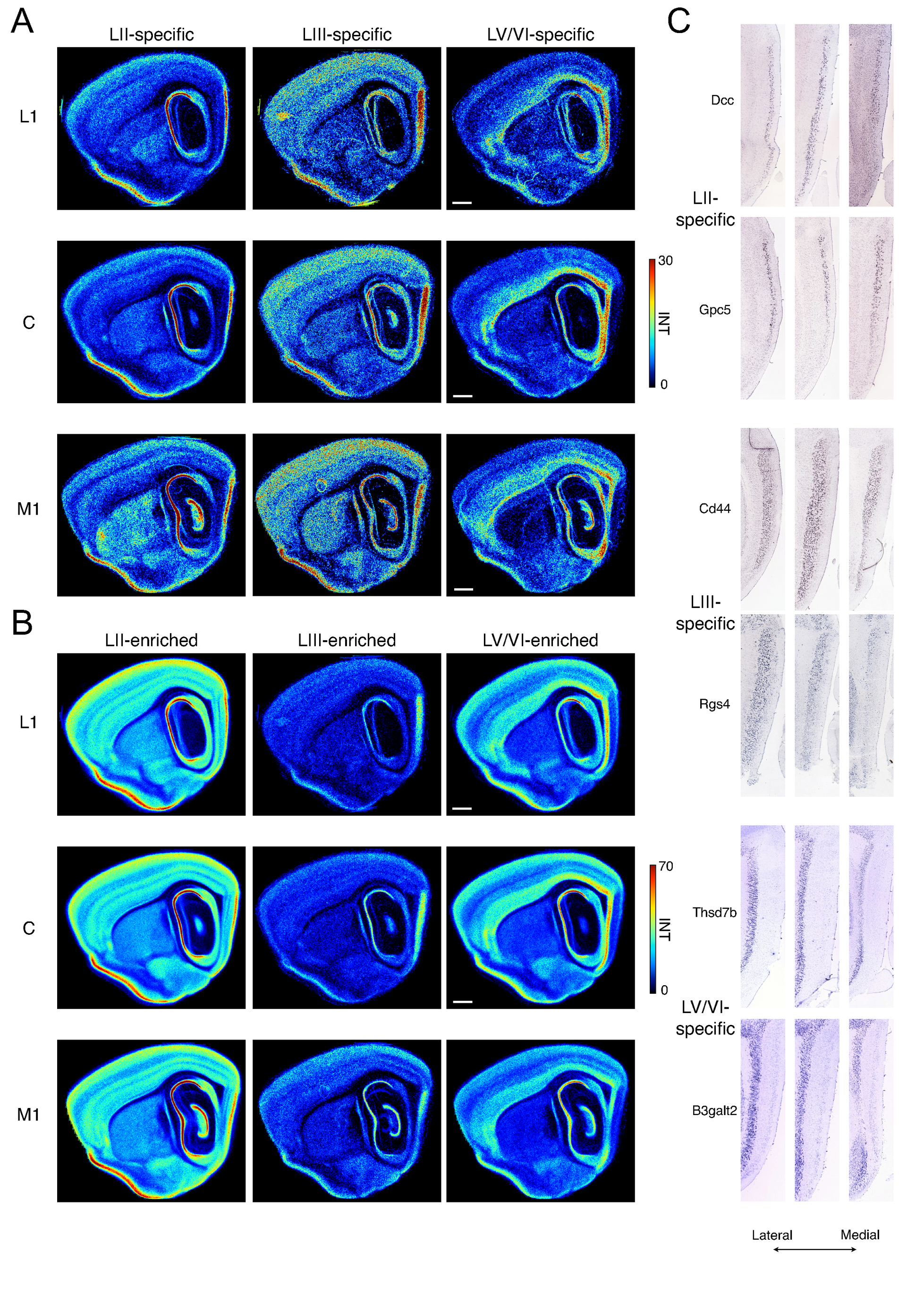

Supplement: S6 Fig — Related to Figs. 4 and 5. (A-B) Images show the mean expression patterns of genes in the re-registered ABA data set that show (A) layer-specific or (B) layer-enriched gene expression in each of the three major laminar regions. Images are shown for the central (C), adjacent lateral (L1) and adjacent medial (M1) sections. (C) Example ISH images at different mediolateral extents are shown for layer-specific genes in layers II, III and V/VI. (TIFF) [file pcbi.1004032.s006.tiff]

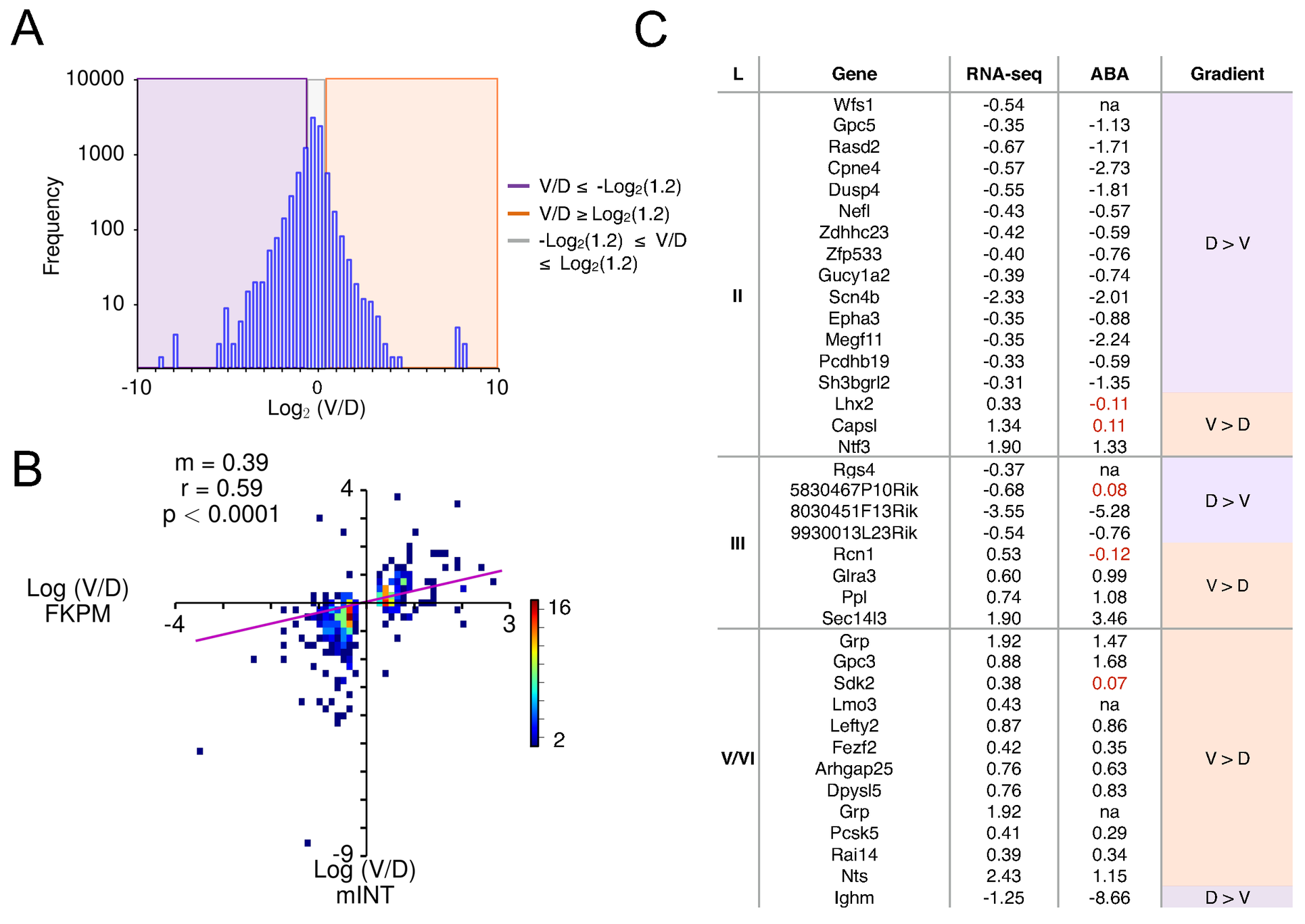

Supplement: S7 Fig — Related to Fig. 7. (A) Histogram shows the distribution of differences in dorsoventral expression (log2 (mINT Ventral / mINT Dorsal)) for all genes (black) with mINT ≥ 2 in the ABA re-registered data set. Colored boxes indicate the scores corresponding to genes classified with differential expression: D>V (purple) or V>D (orange). (B) The log fold change in ABA mINT between ventral and dorsal regions is plotted as a function of log fold change in RNA-Seq mean FPKM for genes found with Cuffdiff 2 to have statistically significant (FDR < 0.05) dorsoventral differences in RNA-Seq mean FPKM. Colors represent number of genes. The linear regression line is indicated in magenta. (C) Table shows dorsoventral differences in ABA and RNA-Seq expression for all layer-specific genes identified as D>V or V>D according to Cuffdiff 2 analysis. (TIFF) [file pcbi.1004032.s007.tiff]
